# Role of the miR-301a/Fra-2/GLIPR1 axis in lung cancer cisplatin resistance

**DOI:** 10.1038/s41392-022-01228-z

**Published:** 2023-01-27

**Authors:** Gian Luca Rampioni Vinciguerra, Marina Capece, Rosario Distefano, Giovanni Nigita, Andrea Vecchione, Francesca Lovat, Carlo M. Croce

**Affiliations:** 1 Department of Cancer Biology and Genetics and Comprehensive Cancer Center, The Ohio State University, Columbus, OH 43210 USA; 2 Department of Clinical and Molecular Medicine, University of Rome ‘Sapienza’, Sant’Andrea Hospital, Rome, 00189 Italy

**Keywords:** Lung cancer

Non-small cell lung cancer (NSCLC) is the leading cause of cancer-related mortality worldwide. Platinum-based chemotherapy represents the main treatment, despite about the 70% of tumors are intrinsically resistant. Deciphering the mechanisms of platinum-resistance is urgently needed.^1^

MiR-301a has been reported to be overexpressed in many tumor types and to play an oncogenic role by modulating several immediate early genes encoding transcription factors.^2^ Despite its pro-tumoral activity is well-documented, a miRNA profiling found that miR-301a is downmodulated in platinum-resistant NSCLC cells.^3^

To elucidate the role of miR-301a in the platinum response of NSCLC, we consulted the putative targets of miR-301a using TargetScan software,^4^ identifying Fos-related antigen-2 (FOSL2/Fra-2), an immediate early gene coding a transcription factor. Fra-2 belongs to the activated protein-1 (AP-1) family and is involved in the mechanisms of resistance to platinum in ovarian cancer^5^ and to anti-EGFR therapy in NSCLC.^6^ Cloning the 3′UTR miRNA-301a-binding sequence of FOSL2/Fra-2 into the psiCHECK-2 vector, we observed that luciferase activity was significantly decreased by miR-301a expression and rescued by miR-301a-binding site deletion (Fig. 1a, supplementary Fig. 1a).

**Fig. 1:**
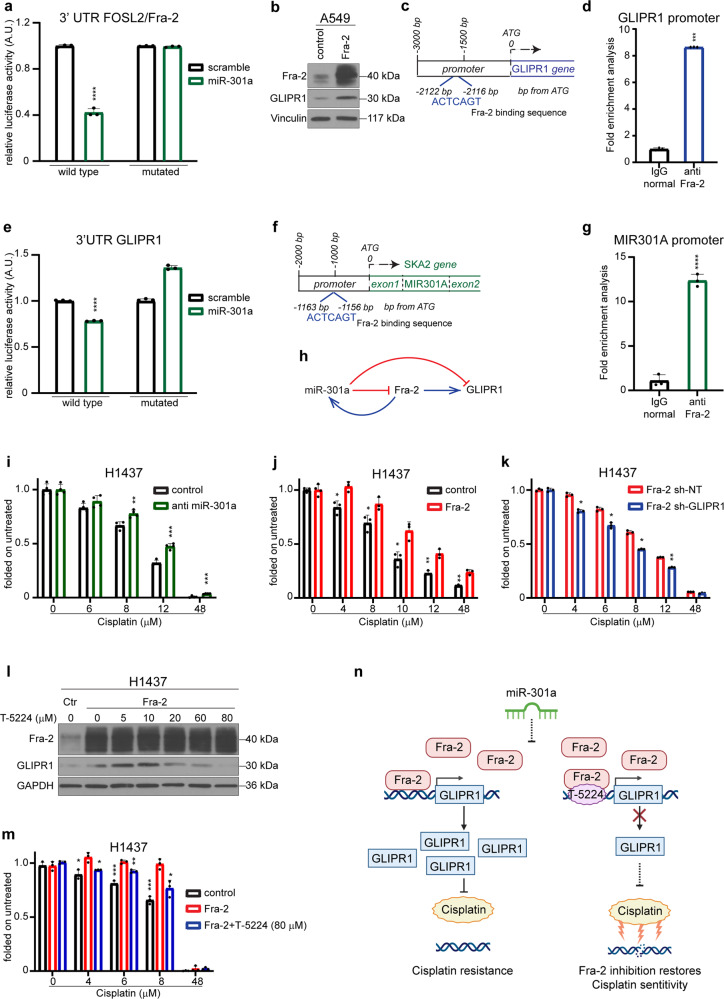
miR-301a/Fra-2/GLIPR1 axis regulates cisplatin resistance in NSCLC. **a** Histogram representing the normalized luciferase activity of psiCHECK2 vector with FOSL2 WT 3′UTR insert and with FOSL2 mutated 3′UTR, containing a deletion of the miR-301a target site in the 3′UTR. Luciferase activity was measured after 24 h post transfection in HEK293 cells. Data represent the mean ± SD from at least three independent experiments and statistical significance was evaluated by Student’s t-test. ****p-value ≤ 0.0001. **b** Western blot analysis of Fra-2 and GLIPR1 in A549 lung cancer cells after transfection or not with Fra-2, as indicated. Vinculin was used as loading control. **c, d** Schematic diagram of Fra-2 binding sequence on GLIPR1 promoter designed for Chromatin immunoprecipitation (ChIP) assay (**c**). Fra-2 bound to the GLIPR1 promoter indicated by ChIP in A549 parental cells (**d**). Data represent the fold enrichment over the IgG control and statistical significance was evaluated by Student’s t-test. ***p-value < 0.001. **e** Histogram representing the normalized luciferase activity of psiCHECK2 vector with GLIPR1 WT 3′UTR insert and with GLIPR1 mutated, containing a deletion of the miR-301a target site in the 3′UTR. Luciferase activity was measured after 24 h post transfection in HEK293 cells. Data represent the mean ± SD from at least three independent experiments and statistical significance was evaluated by Student’s t-test. ****p-value ≤ 0.0001. **f, g** Schematic representation of Fra-binding sequence on MIR301A promoter (**f**). Fra-2 bound to the MIR301A promoter indicated by ChIP analysis in H1437 parental cells (**g**). Data represent the fold enrichment over the IgG control. ****p-value ≤ 0.0001. **h** Schematic graphic of positive feedback loop of miR-301a/Fra-2/GLIPR1 axis. **i–k** MTS cell viability assay of miR-301a silenced (**i**), Fra-2 overexpressing (**j**) and Fra-2 overexpressing/GLIPR-1-silenced (**k**) H1437 cells treated with increasing concentrations of cisplatin. Data are collected after 48 h of cisplatin treatment and are folded over the untreated cells. *p-value < 0.05; **0.001 < *p*-value ≤ 0.01. **l** Western blot analysis of Fra-2 and GLIPR1 in Fra-2 overexpressing H1437 cells treated with increasing doses of T-5224. GAPDH was used as loading control. **m** MTS cell viability assay of parental and Fra-2 overexpressing H1437 cells treated with cisplatin alone or in combination with T-5224 in sequential regimen. Statistical significance was evaluated by Student’s t-test and asterisks mark the statistically differences as follow: *p-value ≤ 0.05. **n** Working model for miR-301a/FOSL2/GLIPR1 axis in the modulation of cisplatin resistance in NSCLC. Downmodulation of miR-301a determines cisplatin resistance via Fra-2 and GLIPR1. Administration of AP-1 inhibitor T-5224 affects GLIPR1 transcription, restoring cisplatin sensitivity

In lung adenocarcinoma cohort from The Cancer Genome Atlas (TCGA) dataset, miR-301a expression was significantly higher in tumors compared to normal tissues (supplementary Fig. 1b). Conversely, FOSL2/Fra-2 was downmodulated in tumors compared to normal samples (supplementary Fig. 1c). In tumor samples, miR-301a inversely correlated with FOSL2/Fra-2 (supplementary Fig. 1d). Altogether, luciferase assay and in silico results supported that Fra-2 is a direct target of miR-301a.

In order to identify which genes could be indirectly targeted by miR-301a via Fra-2 regulation in NSCLC, we investigated the gene expression of A549 cells transfected or not with Fra-2. Filtered results from microarray analysis (*p*-value < 0.0001, Fold-Change > 2.0) (supplementary Table 1) identified 11 genes overexpressed in Fra-2 transfected cells (supplementary Fig. 2a). Considering a positive correlation with FOSL2/Fra-2 (supplementary Fig. 2b) and a negative correlation with miR-301a (supplementary Fig. 2c) in the TCGA dataset, we focused on GLI pathogenesis-related 1 (hereafter, GLIPR1), since its relevant role in the platinum resistance of lung cancer and its documented overexpression in platinum-resistant cells.^7^ Consistently, modulation of Fra-2 altered GLIPR1 expression in A549 cells (Fig. 1b, supplementary Fig. 2d).

To test whether the GLIPR1 upregulation is directly dependent on Fra-2 transcriptional activity, we scanned the 3 kb region upstream of the GLIPR1 start codon, identifying an AP-1 binding sequence known as TPA-responsive element (TRE) motif from -2122 to -2116 bp (Fig. 1c). Thus, we demonstrated that Fra-2 directly bound (by Chromatin immunoprecipitation (chIP)) (Fig. 1d) and activated (by luciferase reporter assay) (supplementary Fig. 2e) GLIPR1 promoter, supporting that GLIPR1 expression is directly controlled by Fra-2.

Moreover, consulting TargetScan, we identified GLIPR1 as a putative target of miR-301a. Then, we validated that miR-301a directly targets 3′UTR seed region of GLIPR1 by luciferase assay (Fig. 1e, supplementary Fig. 2f). Overall, our data demonstrated that GLIPR1 expression is directly and indirectly regulated by miR-301a via targeting Fra-2 transcriptional activity.

To validate in silico findings, we tested the expression of miR-301a, Fra-2 and GLIPR1 in an independent cohort of 24 paired NSCLC samples and their normal counterpart (supplementary Table 2). In 25% of patients, miR-301a levels were higher in NSCLC samples compared to the control tissue (supplementary Fig. 3a). In all tumor samples, we observed a significant anticorrelation between miR-301a and Fra-2/GLIPR1 (supplementary Fig. 3b, c) and, conversely, a significant correlation between Fra-2 and GLIPR1 (supplementary Fig. 3d). Particularly, the fraction of tumor samples with increased levels of miR-301a (red squares in supplementary Fig. 3a) showed a significant downmodulation of Fra-2 and GLIPR1 expression (red squares in supplementary Fig. 3b, c) as also confirmed by western blot analysis (supplementary Fig. 3e).

Next, testing protein levels and gene expression of Fra-2, GLIPR1 and miR-301a in a panel of NSCLC cell lines (supplementary Fig. 4a–d), we identified cell lines with low miR-301a and high Fra-2/GLIPR1 expression (H1975, H647 and H2030 cells) and others with high miR-301a levels and low/no expression of Fra-2/GLIPR1 (H1437, H1299 cells).

In this context, miR-301a had a functional role on the expression of both Fra-2 and GLIPR1, since miR-301a upregulation reduced the expression of its targets (supplementary Fig. 5a–h) and miR-301a silencing significantly increased the expression of Fra-2 and GLIPR1 (supplementary Fig. 5i–n). Likewise, modulation of Fra-2 significantly impacted on GLIPR1 levels: Fra-2 silencing reduced GLIPR1 expression in H1975 (supplementary Figs. 5d, 6a–c), H647 (supplementary Fig. 5f) and H2030 (supplementary Fig. 5h) and Fra-2 overexpression increased GLIPR1 levels in H1437 (supplementary Fig. 6d–f) and H1299 cells (supplementary Fig. 6g–i).

In these settings, we observed that Fra-2 modulation also significantly altered miR-301a levels in all tested cell lines (supplementary Figs. 5e, g, 6c, f, i). This unexpected result suggested that MIR301A gene could be regulated by Fra-2 transcriptional activity.

In support of this, we found that MIR301A is enclosed among the Fra-2 target genes in ChIP-seq analysis on A549 cells by consulting the Harmonizome resource^8^ (FOSL2_A549_hg19_1, ENCODE Transcription Factor Targets dataset^9^). MIR301A is located in the first intron of SKA2 gene and, scanning the 2 kb region upstream of the SKA2 start codon, we identified a TRE motif, AP-1 binding sequence (Fig. 1f). By ChIP assay (Fig. 1g) and by luciferase assay (supplementary Fig. 6j) we confirmed the bond and the transcriptional activity of Fra-2 on MIR301A promoter.

Overall, our results support the occurrence of a positive feedback loop in which miR-301a modulates the expression of GLIPR1 and Fra-2 that, in turn, increases the transcription of MIR301A gene (Fig. 1h).

Further analyzing our results, we noticed that GLIPR1 expression was less affected by miR-301a/Fra-2 axis in H1299 (supplementary Fig. 7a, b), than in H1437 cells (supplementary Fig. 7c, d). Since in other malignancies GLIPR1 is reported to be silenced by methylation, we hypothesized that GLIPR1 expression could be repressed by this mechanism in H1299 cells. In four out of 10 NSCLC lines GLIPR1 methylation was detected (supplementary Fig. 7e). By cloning bisulfite-converted DNA and sequencing analysis, we observed a near-complete, partial, and null methylation of GLIPR1 promoter upstream of transcription start site (TSS) in H1299, H1437 and H1975 DNA, respectively (supplementary Fig. 7f). Thus, treatment with 5-aza-dC increased GLIPR1 expression in H1299 and H1437, but not in H1975 cells (supplementary Fig. 7g–i). These results suggest that in H1299 cells, the methylation status of GLIPR1 could interfere with miR-301a/Fra-2 axis regulation.

Next, we wondered whether miR-301a/Fra-2 axis could modulate cisplatin sensitivity of NSCLC cells and if its activity could be eventually exerted via GLIPR1. Firstly, we assessed that miR-301a silencing and Fra-2 overexpression significantly increased cisplatin-resistance in H1437 cells (Fig. 1i, j), while Fra-2 silencing increased cisplatin-sensitivity in H647 and H2030 cells (supplementary Fig. 8a, b). Intriguingly, in cisplatin-treated H1299 cells, in which GLIPR1 promoter was hypermethylated, cell viability was not affected by Fra-2 overexpression (supplementary Fig. 8c). Conversely, ectopic introduction of GLIPR1 strongly induced cisplatin resistance (supplementary Fig. 8d).

Then, we observed that both GLIPR1 silencing and GLIPR1 overexpression restored cisplatin sensitivity of Fra-2 overexpressing H1437 cells (Fig. 1k, supplementary Fig. 8e) and Fra-2 silenced H2030 cells (supplementary Fig. 8f), respectively. Altogether, our results confirmed that Fra-2 regulation on chemoresistance is specifically mediated by GLIPR1.

Then, we wondered whether administration of a selective AP-1 inhibitor^10^ (T-5224) could represent a valuable strategy to inhibit Fra-2 transcriptional activity and eventually counteract cisplatin-resistance in NSCLC. Increasing concentrations of T-5224 reduced GLIPR1 levels in Fra-2 overexpressing H1437 cells (Fig. 1l, supplementary Fig. 9a, b), without affecting their viability (supplementary Fig. 9c). Accordingly, T-5224 administration impinged on Fra-2 chromatin-binding capability with GLIPR1 promoter by ChIP analysis, strongly inhibiting Fra-2 transcriptional activity (supplementary Fig. 9d).

Next, testing different administration of T-5224 (T) and cisplatin (C) (T → C regimen, supplementary Fig. 9e and T + C regimen, supplementary Fig. 9f, g), we found that cisplatin sensitivity was significantly increased by pre-treatment with T-5224 (T → C regimen) in Fra-2 overexpressing H1437 cells, partially restoring the phenotype observed in control cells (Fig. 1m). By contrast, T-5224 did not alter cisplatin sensitivity of H1437 parental cells (supplementary Fig. 9h) and Fra-2 overexpressing H1299 cells (supplementary Fig. 9i), suggesting that the effect of T-5224 was specifically dependent on Fra-2 overexpression and its activity on GLIPR1 promoter.

Collectively, we present compelling evidence that miR-301a regulates GLIPR1 expression directly and indirectly via Fra-2 in NSCLC. We firstly demonstrated that the novel miR-301a/Fra-2/GLIPR1 axis contributes to cisplatin resistance. Our study also provides a clear rationale for the development of a combined schedule with Fos/AP-1 inhibitors and cisplatin in lung cancer (Fig. 1n), supporting that miR-301a/Fra-2/GLIPR1 expression may serve as a biomarker to stratify patients who better respond to cisplatin alone or in combination with Fos/AP-1 inhibitors.

## Supplementary information

Supplementary material

Supplementary Table 1 List of differentially expressed genes in A549 cells transfected or not with Fra-2

original and uncropped films of western Blot

## Data Availability

All raw data that support the findings of this study have been submitted to the NCBI Gene Expression Omnibus (GEO; https://www.ncbi.nlm.nih.gov/geo/) under accession number GSE213816. The gene and miRNA Level 3 expression data were retrieved for GDC data portal: https://portal.gdc.cancer.gov. All data reported in this paper are available upon request.
