# Supplementary material for: Role of the miR-301a/Fra-2/GLIPR1 axis in lung cancer cisplatin resistance
Source: Signal Transduct Target Ther. 2023 Jan 27;8:37. doi: 10.1038/s41392-022-01228-z (PMC9879967; doi:10.1038/s41392-022-01228-z)
Supplement: Supplementary file 1 — Supplementary material [file 41392_2022_1228_MOESM1_ESM.docx]

Supplementary Materials for

**Role of the miR-301a/Fra-2/GLIPR1 axis in lung cancer cisplatin resistance**

Gian Luca Rampioni Vinciguerra^1^, Marina Capece^1^, Rosario Distefano^1^, Giovanni Nigita^1^, Andrea Vecchione^2^, Francesca Lovat^1^ and Carlo M. Croce^1^.

1. Department of Cancer Biology and Genetics and Comprehensive Cancer Center, The Ohio State University, Columbus, OH, USA, 43210.
2. Department of Clinical and Molecular Medicine, University of Rome 'Sapienza', Sant'Andrea Hospital, Rome, Italy, 00189.

**Corresponding Authors:** Francesca Lovat, 1070 Biomedical Research Tower, 460 W 12^th^ Avenue, Columbus 43210 OH USA; email: [francesca.lovat@osumc.edu](mailto:francesca.lovat@osumc.edu) and Carlo M. Croce, 1082 Biomedical Research Tower, 460 W 12^th^ Avenue, Columbus 43210 OH USA; Phone: (+1) 614-292-4930; Fax (+1) 614-292-3358; email: [carlo.croce@osumc.edu](mailto:carlo.croce@osumc.edu)

**This PDF file includes:**

Supplementary text

Supplementary Fig S1-S9

Supplementary Table S2-S3.

**Other Supplementary Materials for this manuscript include the following:**

Supplementary Table S1

**SUPPLEMENTARY TEXT**

**ABSTRACT**

Cisplatin-based therapy represents the main treatment for lung cancer. However, the majority of tumors is intrinsically resistant. By investigating miR-301a role in lung cancer, we found that miR-301a regulates GLIPR1 expression, a mediator of cisplatin resistance, directly and indirectly *via* the transcription factor Fos-related antigen-2 (FOSL2/Fra-2). This novel miR-301a/Fra-2/GLIPR1 axis is autoregulated by feedback loop where Fra-2 also promotes the transcription of MIR301A gene.

In an internal cohort of lung cancer samples, the overexpression of miR-301a identified a fraction of tumors with low expression of Fra-2 and GLIPR1.

In lung cancer cells, miR-301a/Fra-2 contributed to cisplatin resistance by regulating GLIPR1 expression; thus, administration of Fos/AP-1 inhibitor significantly increased cisplatin sensitivity.

Overall, our findings support that miR-301a/Fra-2/GLIPR1 axis contributes to cisplatin resistance in lung cancer and could serve as biomarker to stratify patients who may benefit from cisplatin administration, alone or in combination with Fos/AP-1 inhibitors.

**Statement of Significance**

We identified and characterized this novel miR-301a/Fra-2/GLIPR1 axis as a potential biomarker of cisplatin response in lung cancer. This axis may identify a fraction of lung cancer patients for whom the cisplatin administration alone is no more effective and needs new combination therapies.

**INTRODUCTION**

Lung cancer is the leading cause of cancer-related mortality in both men and women worldwide ^1^. Among the histological subtypes of lung cancer, non-small cell lung cancer (NSCLC) accounts for the 85% of cases ^2^. In the past decades, significant advances in the understanding of molecular pathogenesis of NSCLC have led to identify targetable mutations with a significant impact on the diagnosis, prognosis and treatment of these patients ^3,4^. Despite the fact that these improvements have drastically changed the clinical approach to NSCLC, cytotoxic platinum-based chemotherapy still represents the cardinal treatment for the majority of patients ^2^. Nevertheless, about the 70% of NSCLC are intrinsically resistant to platinum compounds and this strongly limits the response rate to therapy, explaining the high lethality of the disease ^5^.

Therefore, a deeper investigation of the molecular mechanisms of chemoresistance is urgently needed to improve the clinical outcome of patients with NSCLC.

MiR-301a is a member of the miR-130 family and its overexpression has been reported in several tumor types including breast ^6^, prostate ^7^, glioblastoma ^8^, pancreatic ^9,10^ and lung cancer ^11–13^.

In NSCLC, miR-301a promotes anchorage-independent growth ^12^, cell migration, metastatic dissemination ^11^ and impacts on tumor microenvironment stimulating a pro-tumorigenic inflammatory status ^13^. Moreover, miR-301a affects response to treatments, conferring resistance to trastuzumab in HER2-positive gastric cancer ^14^ and to radiotherapy in glioblastoma ^8^. Considering NSCLC, a miRNA profiling found that miR-301a is downmodulated in platinum-resistant cells ^15^. Intriguingly, in both malignant and tumor-associated cells, miR-301a mostly acts by modulating immediate early genes encoding transcription factors ^13,16,17^.

In lung and pancreatic cancer, by targeting NF-kB-repressing factor (NKRF), miR-301a potently activates NF-kB that, in turn, regulates the transcription of miR-301a, in positive regulatory feedback that maintains NF-kB active ^10^. In the microenvironment, miR-301a indirectly activates STAT3 transcriptional activity, favoring tumor cell proliferation ^13^, pulmonary fibrosis ^16^ and modulation of inflammatory T cells ^18^.

In this study, we demonstrate that in NSCLC miR-301a directly targets Fos-related antigen-2 (hereafter, Fra-2), an immediate early gene belonging to the activated protein-1 (AP-1) transcription factor family.

Fra-2 is well known to regulate the composition of the extracellular matrix in physiological processes; however, its role in cancer appears more elusive. Recent evidence indicates that, while Fra-2 overexpression increases the metastatic potential of breast cancer cells ^19^, its downmodulation promotes migratory and invasive potential of melanoma cells ^20^. In lung cancer, the role of Fra-2 still needs to be elucidated. Fra-2 expression positively correlates with tumor metastasis and poor prognosis in NSCLC patients ^21–23^. In contrast, Fra-2 seems to play a very limited role during the lung tumorigenesis: in fact, its activation is not triggered by carcinogen like cigarette smoking that, conversely, induces other members of the AP-1 family ^24,25^, thus indicating that the Fra-2 role is likely both tumor- and context-dependent. As miR-301a, also Fra-2 is involved in the mechanisms of drug response; in particular, Fra-2 overexpression confers resistance to platinum in ovarian cancer ^26^ and to anti-EGFR therapy in NSCLC ^27^.

Here, by investigating both the involvement of miR-301a in the Fra-2 regulation and Fra-2 transcriptional activity in NSCLC, we identify a new mechanism through which miR-301a directly and indirectly inhibits GLIPR1 expression *via* Fra-2. Then, considering that both Fra-2 and GLIPR1 modulate cisplatin resistance ^26,28–30^, we investigate the role of the newly identified miR-301a/Fra-2/GLIPR1 axis in NSCLC response to cisplatin.

**RESULTS**

**miR-301a targets Fra-2 and inversely correlates with its expression in lung adenocarcinoma**

After consultation of a computational target prediction algorithms (TargetScan) ^31^, we identified Fos-related antigen-2 (FOSL2/Fra-2) as an immediate early gene coding a transcription factor, among the putative targets of miR-301a. In literature, it is reported that miR-301a exerts an oncogenic role in different tumors, modulating the expression of different transcription factors ^10,17^. To dissect the possible role of miR-301a in the regulation of its putative target Fra-2 coding gene, FOSL2, we firstly assessed their expression in The Cancer Genome Atlas (TCGA) dataset. miR-301a expression was significantly increased in lung adenocarcinoma samples respect to the normal tissues (supplementary Fig 1b). In contrast, Fra-2 was downmodulated in tumors compared to controls (supplementary Fig 1c).

Focusing on the tumor samples, levels of miR-301a had a significant inverse correlation with Fra-2 expression (supplementary Fig 1d). This evidence suggested the possibility that miR-301a could regulate Fra-2 in lung adenocarcinomas and their expression could stratify these tumors in two different subgroups characterized by high-miR-301a/low-Fra-2 and low-miR-301a/high-Fra-2, respectively (supplementary Fig 1d). To determine whether Fra-2 is a direct target of miR-301a, we cloned the 3’­UTR miRNA-binding sequence of the gene into the multiple cloning region of the psiCHECK-2 vector and measured the luciferase activity. The expression of miR-301a significantly decreased luciferase activity (Figure 1a, supplementary Fig 1a). Furthermore, when luciferase assay was performed on a construct deleted for miR-301a binding site, miR-301a expression did not alter the luciferase activity (Figure 1a, supplementary Fig 1a). Altogether, luciferase assay and *in silico* results supported that Fra-2 is a direct target of miR-301a in lung adenocarcinoma.

**Fra-2 transcriptional activity regulates GLIPR1 expression in lung cancer**

Data collected herein before indicated that Fra-2 expression is decreased in lung adenocarcinoma samples compared to the normal tissue in the TCGA dataset.

We thus proceeded to investigate which genes are regulated at transcriptional level by Fra-2, in order to identify possible indirect targets of miR-301a in NSCLC.

To this aim, we performed a microarray analysis on A549 lung cancer cells transfected or not with Fra-2 and investigated their gene expression. We identified 416 genes differentially expressed between the two conditions (p-value<0.01, Fold-Change>1.5) (supplementary Fig 2a). Then, we focused on the genes upregulated in Fra-2 overexpressing cells respect to the control and, among them, we selected 65 genes having a fold-change higher than 2.0 (supplementary Table 1).

By applying a more stringent inclusion criterion (p-value<0.0001), we finally identified 11 genes overexpressed by Fra-2. Considering a positive correlation with Fra-2 (supplementary Fig 2b) and a negative correlation with miR-301a (supplementary Fig 2c) in the TCGA dataset, we focused on GLI pathogenesis-related 1 (hereafter, GLIPR1), since its relevant role in the platinum resistance ^28–30^. In fact, GLIPR1 has been shown to play a dual role in NSCLC: on one hand, GLIPR1 has been reported to be downregulated during lung tumorigenesis ^32^; on the other hand, GLIPR1 mediates cisplatin sensitivity and is overexpressed in cisplatin-resistant cells ^28,29^. However, the mechanism of GLIPR1 regulation has not been clarified yet. Consistent with microarray results, modulation of Fra-2 altered GLIPR1 expression in A549 cells by western blot analysis (Figure 1b, supplementary Fig 2d).

In order to test whether GLIPR1 upregulation observed in Fra-2 overexpressing cells was directly dependent on Fra-2 transcriptional activity, we scanned the 3kb region upstream of the GLIPR1 start codon, identifying an AP-1 binding sequence known as TPA-responsive element (TRE) motif ^33^ from -2122 to -2116 bp (Figure 1c). Thus, we demonstrated that Fra-2 directly bound (by Chromatin immunoprecipitation (chIP)) (Figure 1d) and activated (by luciferase reporter assay) (supplementary Fig 2e) GLIPR1 promoter, supporting that GLIPR1 expression is directly controlled by Fra-2.

Moreover, consulting TargetScan ^31^, we identified GLIPR1 as a putative target of miR-301a. Then, we tested whether miR-301a could also directly regulate GLIPR1 expression acting at its 3’UTR. Luciferase assay in HEK293 cells demonstrated that miR-301a overexpression significantly reduced the luciferase activity and that, site-directed mutagenesis of the miRNA seed region completely restored luciferase activity observed in control cells (Figure 1e, supplementary Fig 2f).

Overall, our data demonstrated that GLIPR1 expression is directly regulated by miR-301a and indirectly by miR-301a-targeted Fra-2 transcriptional activity.

**miR-301a inversely correlates with Fra-2 and GLIPR1 in an independent cohort of NSCLC patients**

To validate *in silico* and *in vitro* findings, we tested the expression of miR-301a, Fra-2 and GLIPR1 by qRT-PCR in an independent cohort of 24 paired NSCLC samples and their normal counterpart (supplementary Table 2). In 25% of patients, miR-301a levels were higher in NSCLC samples compared to the control tissue (supplementary Fig 3a). Next, we determined the correlation between miR-301a and its targets among the tumor samples. In line with the observation from the TCGA dataset, we reported a significant anticorrelation between miR-301a and Fra-2/GLIPR1 (supplementary Fig 3b, 3c) and, conversely, a significant correlation between Fra-2 and GLIPR1 (supplementary Fig 3d). Particularly, the fraction of tumor samples with increased levels of miR-301a (red squares in supplementary Fig 3a) showed a significant downmodulation of Fra-2 and GLIPR1 expression (red squares in supplementary Fig 3b, 3c). Aiming to verify if this reduction of transcript expression was also reflected on protein levels, we tested Fra-2 and GLIPR1 expression in patients’ samples with low miR-301a expression and high miR-301a expression (red squares in supplementary Fig 3a). Our results clearly demonstrated that miR-301a overexpressing tumors showed a reduced expression of Fra-2 and GLIPR1 compared to miR-301a low-expressing tumors by western blot analysis (supplementary Fig 3e).

**Mir-301a regulation occurs as a positive feedback loop *via* Fra-2 in NSCLC cell lines**

Next, we tested protein levels of Fra-2 and GLIPR1 and the expression of their relative genes in a panel of 10 NSCLC cell lines (supplementary Fig 4a-d). Protein levels were variable among the different cells, identifying some of them highly expressing (H1975, H647 and H2030 cells) and others with low/no expression (H1437 and H1299 cells) of both Fra-2 and GLIPR1. Interestingly, levels of miR-301a were consistently higher in H1437 and H1299 respect to H1975, H647 and H2030 cells, despite this inverse correlation between Fra-2/GLIPR1 and miR-301a was not extendable to all NSCLC cell lines (supplementary Fig 4a, 4d). To better understand the functional role of miR-301a in both Fra-2 and GLIPR1 expression, we modulated miR-301a expression in different cell lines. The silencing of miR-301a increased both Fra-2 and GLIPR1 levels in H1437 (supplementary Fig 5i-k) and H1299 cells (supplementary Fig 5l-n), while miR-301a overexpression significantly reduced Fra-2 and GLIPR1 expression in H1975 (supplementary Fig 5a-d), in H647 (supplementary Fig 5e, 5f) and in H2030 (supplementary Fig 5g, 5h) cells.

Likewise, alteration of Fra-2 levels significantly impacted on GLIPR1 expression: in fact, Fra-2 overexpression increased GLIPR1 levels in both H1437 (supplementary Fig 6d, 6e) and H1299 cell lines (supplementary Fig 6g, 6h), while Fra-2 silencing reduced GLIPR1 in H1975 (supplementary Fig 5d, 6a, 6b), in H647 (supplementary Fig 5f) and in H2030 (supplementary Fig 5h) cells. Intriguingly, in these settings, we observed that modulation of Fra-2 also induced a significant alteration of miR-301a levels in all tested cell lines (supplementary Fig 5e, 5g, 6c, 6f, 6i). This unexpected result suggested that Fra-2 transcriptional activity could also regulate MIR301A gene.

Then, by consultation of the Harmonizome resource^34^, we assessed that MIR301A gene is enclosed among the target genes of Fra-2 obtained by ChIP-seq analysis on A549 lung cancer cell lines (FOSL2_A549_hg19_1, ENCODE Transcription Factor Targets dataset^35^). MIR301A is located in the first intron of SKA2 gene. Then, we scanned the 2kb region upstream of the SKA2 start codon, identifying a TRE motif, AP-1 binding sequence from -1163 to -1156 bp (Figure 1f). By ChIP assay (Figure 1g) and by luciferase assay (supplementary Fig 6j), we confirmed the bond and the transcriptional activity of Fra-2 on MIR301A promoter in H1437 cells.

Overall, our results support the occurrence of a positive feedback loop in which miR-301a modulates the expression of GLIPR1 and Fra-2 that, in turn, increases the transcription of the MIR301A gene (Figure 1h).

**Promoter methylation suppresses GLIPR1 expression induced by Fra-2 transcriptional activity in NSCLC**

Next, we analyzed the impact of miR-301a silencing and Fra-2 overexpression on GLIPR1 protein levels, in H1437 and H1299 cell lines where GLIPR1 is low or not expressed (supplementary Fig 4a). In H1437 cells, both miR-301a silencing and Fra-2 overexpression (supplementary Fig 7c, 7d) strongly increased GLIPR1 levels compared to controls. However, in H1299 cells GLIPR1 expression was induced less efficiently by miR-301a silencing compared to Fra-2 overexpression (supplementary Fig 7a, 7b). These results were consistent with the data collected by qRT-PCR assay where a small increase of Fra-2 level was necessary to overexpress GLIPR1 (supplementary Fig 6d, 6e) in H1437 cells, while a more pronounced overexpression of Fra-2 was required to activate GLIPR1 transcription in H1299 cells (supplementary Fig 6g, 6h).

Collectively, our data indicated that GLIPR1 expression is tightly dependent on miR-301a/Fra-2 axis in some NSCLC models (e.g., H1437 cell line), while in others (e.g., H1299 cell line) different mechanisms could be involved. Since in H1299 cells GLIPR1 expression was more induced by Fra-2 overexpression, that acts at transcriptional level, than through miR-301a silencing (supplementary Fig 7a, 7b), that works post-transcriptionally, we speculated that these mechanisms are likely connected with the transcription of GLIPR1 gene. In other malignancies, GLIPR1 is reported to be silenced by methylation of its promoter ^36,37^. We analyzed the methylation status of GLIPR1 promoter in bisulfite-converted DNA from 10 NSCLC cell lines using methylation-specific PCR. Methylation of GLIPR1 was detected in four out of 10 analyzed cell lines (supplementary Fig 7e). In particular, the methylation of GLIPR1 promoter was further assessed by cloning bisulfite-converted DNA from H1437, H1299 and H1975 cells and by sequencing analysis of GLIPR1 promoter from individual bacterial colonies. Analysis of five clones revealed a near-complete, partial and null methylation of GLIPR1 promoter upstream of TSS in H1299, H1437 and H1975 DNA, respectively (supplementary Fig 7f). To confirm the bisulfite sequencing, we treated these cell lines with the 5-aza-dC demethylating drug to determine whether the GLIPR1 gene may be reactivated after demethylation. Treatments with 5-aza-dC increased expression of GLIPR1 in both H1299 and H1437, but not in H1975 cell lines (supplementary Fig 7g-i), suggesting that, in this setting, the methylation status of GLIPR1 could interfere with miR-301a/Fra-2 axis regulation.

**Fra-2 expression modulates cisplatin sensitivity *via* GLIPR1 in NSCLC**

Data so far pointed to a role of miR-301a/Fra-2 axis in the regulation of GLIPR1, at least in NSCLC cell lines whose GLIPR1 promoter is not methylated. As mentioned before, miR-301a is downmodulated in cisplatin-resistant NSCLC cells ^15^, while Fra-2 and GLIPR1 have been associated with cisplatin resistance in cancer ^26,28–30^. Thus, we tested whether the expression of miR-301a/Fra-2 could also modulate cisplatin sensitivity of NSCLC cells and if their activity could be eventually exerted *via* GLIPR1.

Using H1437 cells, we observed that miR-301a silencing and Fra-2 overexpression significantly increased cisplatin-resistance compared to the control (Figure 1i, 1j). By contrast, Fra-2 silencing significantly increased cisplatin sensitivity in H647 and H2030 cells (supplementary Fig 8a, 8b). Intriguingly, in cisplatin-treated H1299 cells, in which GLIPR1 promoter is methylated, Fra-2 overexpression did not affect cell viability (supplementary Fig 8c), but ectopic introduction of GLIPR1 significantly increased cisplatin resistance (supplementary Fig 8d).

Then, we observed that both GLIPR1 silencing and GLIPR1 overexpression restored cisplatin sensitivity of Fra-2 overexpressing H1437 cells (Figure 1k, supplementary Fig 8e) and Fra-2 silenced H2030 cells (supplementary Fig 8f). Altogether, our results confirmed that Fra-2 regulation on chemoresistance is specifically mediated by GLIPR1.

Recently, the administration of T-5224, a selective inhibitor of Fos/AP1 transcriptional activity, has been reported to be effective in the treatment of both benign and malignant diseases ^38,39^. Moreover, different studies support the possibility that T-5224 may improve the efficacy of other antineoplastic agents when administrated in combination therapy ^40,41^. Considering the role of Fra-2/GLIPR1 axis in cisplatin-sensitivity, we wondered whether the use of Fos/AP-1 inhibitors could represent a valuable strategy to increase the efficacy of cisplatin in NSCLC cells.

To verify this hypothesis, we selected increasing doses of T-5224 that did not affect cell viability of Fra-2 overexpressing H1437 cells (supplementary Fig 9c). Even though T-5224 did not interfere with Fra-2 overexpression (supplementary Fig 9a), levels of GLIPR1 strongly decreased under T-5224 treatment in a dose-dependent manner (Figure 1l, supplementary Fig 9b). Accordingly, ChIP experiments showed that Fra-2 bound more to the GLIPR1 promoter in miR-301a-silenced cells comparing to control. However, T-5224 administration impinged on Fra-2 chromatin-binding capability, acting as a strong negative regulator of Fra-2 transcriptional activity (supplementary Fig 9d).

Next, we tested the combination of low doses of T-5224 (T) and cisplatin (C) on NSCLC cell viability, evaluating both the sequential (T→C regimen, supplementary Fig 9e) and the concomitant administration of these drugs (T+C regimen, supplementary Fig 9f). When these two compounds were jointly administrated (T+C regimen), Fra-2 inhibition did not improve cisplatin cytotoxicity in Fra-2 overexpressing H1437 cells (supplementary Fig 9g). However, when Fra-2 overexpressing cells were pre-treated with T-5224 (T→C regimen), they showed a higher cisplatin sensitivity, partially restoring the phenotype observed in control cells (Figure 1m). Moreover, T→C regimen did not alter cisplatin sensitivity of H1437 parental cells (supplementary Fig 9h) and Fra-2 overexpressing H1299 cells (supplementary Fig 9i), suggesting that the effect of T-5224 was specifically dependent on overexpression and activity of Fra-2 on GLIPR1 promoter.

Overall, our results support that miR-301a/Fra-2 axis contributes to cisplatin resistance *via* GLIPR1, and Fra-2 inhibition increases cisplatin cytotoxicity in Fra-2 overexpressing NSCLC cells.

**DISCUSSION**

Emerging evidence shows that dysregulation of miR-301a is a common event in tumor progression. Intriguingly, miR-301a overexpression exploits an oncogenic function modulating the expression of several immediate early genes and transcription factors like HIF1 ^42^, NF-kB ^10^, Runt-related transcription factor-3 ^11^, STAT3 ^17^ and beta-catenin ^8^. Here, investigating the role of miR-301a in NSCLC, we showed that miR-301a plays a central role in the control of Fra-2 transcriptional activity and consequently of GLIPR1 expression. We demonstrated not only that miR-301a targets Fra-2, thus repressing transcription of GLIPR1 gene, but also directly targets GLIPR1. Moreover, our data support a feedback regulation of miR-301a/Fra-2/GLIPR1 axis, relying on Fra-2-driven transcription of MIR301A gene.

As reported in prostate cancer cells ^36^ and acute myeloid leukemia cells ^37^, GLIPR1 expression is also controlled by the methylation status of its promoter. Here, we showed that GLIPR1 expression is silenced regardless Fra-2 activity/up-regulation, when GLIPR1 promoter is hyper-methylated (e.g., H1299 cell line).

The analysis of lung adenocarcinomas from the TCGA database showed that miR-301a is significantly increased in tumor samples respect to the normal tissue. Among tumor samples, we observed a significant anticorrelation between miR-301a and Fra-2/GLIPR1 and, conversely, a significant correlation between Fra-2 and GLIPR1. Importantly, we confirmed these results in an internal cohort of paired normal and tumor samples from NSCLC patients, observing that 25% of tumors were characterized by miR-301a overexpression. This fraction of miR-301a high-expressing tumors showed significant low levels of both Fra-2 and GLIPR1.

The fact that, in our model, miR-301a induces cisplatin sensitivity by Fra-2 targeting seems to be in contrast with the established knowledge that miR-301a acts as an oncomiR and confers drug resistance to different therapeutics. In fact, miR-301a dysregulation mediates trastuzumab resistance by indirect activation of receptor tyrosine kinases in HER2-positive gastric cancer ^14^ and reduces radiosensitivity in glioblastoma cells *via* Wnt/β-catenin pathway ^8^. However, miR-301a downmodulation has been described in cisplatin refractory NSCLC cells ^15^. Significantly, also Fra-2 overexpression leads to cisplatin resistance in ovarian cancer cells ^26^. Noteworthy, in NSCLC Fra-2 overexpression is a negative prognostic factors, contributing to tumor microenvironment remodeling, to metastatic dissemination ^22^ and to anti-EGFR therapy resistance ^27^. Taken together, these data lead us to speculate that miR-301a/Fra-2 axis may affect cisplatin response in NSCLC. Aiming to identify downstream effectors of Fra-2 transcriptional activity, we focused on GLIPR1 as an attractive candidate since its emerging role in NSCLC. It has been reported that GLIPR1 could act as an antitumor player through inhibiting cancer cell growth ^32^ and as a oncoprotein by inducing cisplatin-resistance ^29,30^.

Validating the role of miR-301a/Fra-2/GLIPR1 axis in the cisplatin-resistance, we also explored the possible combination of T-5224, a Fos/AP-1 inhibitor, to improve cisplatin cytotoxicity. We observed that T-5224 potently counteracted the expression of GLIPR1 in a dose-dependent manner and that pre-treatment with T-5224 improved the efficacy of cisplatin in Fra-2 overexpressing cells. On the other hand, concomitant administration of T-5224 and cisplatin results ineffective, suggesting that T-5224 did not directly improve the cytotoxic activity of cisplatin. According to our findings, the effect of Fra-2 inhibition on cisplatin response is specifically mediated by GLIPR1. In literature, GLIPR1 has been reported to protect cells by DNA-damaging agents and be involved in Bcl-2 upregulation ^29,43^. Therefore, low GLIPR1-expressing cells are more sensitive to cisplatin. In this context, we speculate that applying Fra-2 inhibitor directly before cisplatin efficiently may cooperate in inducing apoptosis due to the role of GLIPR1 in Bcl-2 upregulation.

Moreover, T-5224 acts on whole Fos/AP-1 complex. The opposite effects of the two administration regimens suggest that other Fos proteins may play different and adverse functions respect to Fra-2. In fact, AP-1 is involved in many cellular processes, including cell death ^44,45^. In ovarian cancer patients, downmodulation of c-Fos has been correlated with increased resistance to chemotherapy and shorter survival ^46^.

Further studies are needed to address the precise role of AP-1 heterodimers in lung cancer evolution and drug response to assess the proper combination and timing of targeted therapies.

Taking into account these limitations, our study provides a clear rationale for the development of a combined schedule with Fos/AP-1 inhibitors and cisplatin in lung cancer, supporting that miR-301a/Fra-2/GLIPR1 expression may serve as a biomarker to stratify patients who better respond to cisplatin alone or in combination with Fos/AP-1 inhibitors.

**MATERIALS AND METHODS**

**Human tissue samples**

Twenty-four lung cancer samples with matching NAT (normal adjacent tissue) were obtained from the Tissue Bank of The Ohio State University, under the universal consenting and biobanking protocol, Total Cancer Care (TCC). All patients have provided informed consent for tumor collection, storage, and analysis. The tumor samples in this study were provided as unidentified samples.

Frozen tissues were dissected on dry ice into smaller pieces, in order to perform protein and RNA extraction.

**Cell culture, transfection and reagents**

Lung cancer cell lines H838 (NCI-H838 CRL-5844), H1437 (NCI-H1437 CRL-5872), H460 (NCI-H460 HTB-177), H1299 (NCI-H1299 CRL-5803), A549 (A549 CRM-CCL-185), H2030 (NCI-H2030 CRL-5914), H1563 (NCI-H1563 CRL-5875), H1975 (NCI-H1975 [H-1975, H1975] CRL-5908), H522 (NCI-H522 CRL-5810) and H647 (NCI-H647 CRL-5834), kidney embryonic cells HEK293 (293 [HEK-293] CRL-1573) were purchased from American Type Culture Collection (ATCC) and cultured in RPMI1640 medium (Sigma) supplemented with 10% fetal bovine serum (FBS, Sigma) and 1% Streptomycin/ampicillin solution (Sigma).

For transient transfection, pCMV6-Entry-Fra2 (Myc-DDK-tagged CAT#: RC204146), pCMV6-Entry-GLIPR1 (Myc-DDK-tagged CAT#: RC216882) and pCMV6-Entry (Myc-DDK-tagged CAT#: PS100001) as control (ORIGENE, Rockville, MD) were used for Fra-2 overexpression. FOSL2 siRNA (Product no. EHU072911), GLIPR1 siRNA (Product no. EHU022591) and control siRNA (scramble oligonucleotides; Product no. SIC001) were purchased from Sigma (Mission esiRNA). Pre-miR-301a precursor (Assay ID: PM10978), pre-miR Precursor Negative Control #2 (Cat# AM17111), anti-miR-301a inhibitor (Assay ID: AM10978) and anti-miR Inhibitor Negative Control #1 (Assay ID: AM17010) were obtained from ThermoFisher Scientific. Lipofectamine 2000 (ThermoFischer Scientific) transfection system was used following the manufacturer’s instructions to overexpressing or silencing different genes in A549, H1437, H1299, H2030, H647 and H1975 cell lines.

Cisplatin and T-5224 (c-Fos/activator protein (AP)-1 inhibitor) were purchased from Selleckchem.

**Chromatin immunoprecipitation (ChIP) assay**

SimpleChIP Enzymatic Chromatin ImmunoPrecipitation Kit (Magnetic Beads, Cat.9005S, Cell Signaling Technology) was used following the manufacture’s protocol. The obtained chromatin samples were incubated at 4°C overnight with the following antibodies: normal rabbit IgG (Cat.2729, Cell Signaling technology), anti–Fra-2 (clone D2F1E, Cat.19967, Cell Signaling Technology) and anti-H3 (Cat.4620, Cell Signaling Technology) as positive control. Immunoprecipitated chromatin was purified and analyzed by the real-time quantitative PCR using SimpleChIP Universal qPCR Master Mix (#88989, Cell Signaling Technology). Data were analyzed with the fold enrichment analysis method. Primers used to amplify the indicated GLIPR1 and MIR301A promoters are listed in supplementary Table 3.

**Dual-Luciferase reporter assay**

The predicted miR-301a-binding sites of FOSL2 and GLIPR1 3’UTR were amplified by PCR using specific primers. PCR products were digested with XhoI and NotI (New England Biolabs) and cloned downstream of Renilla luciferase gene of psiCHECK2 vector (Promega). The mutants of FOSL2 and GLIPR1 3’UTR were generated using QuikChange II XL Site-Directed Mutagenesis Kit (Agilent), according to the manufacturer’s protocol. HEK293 cells were co-transfected with 1 μg of psiCHECK2 constructs and 100 nM of pre-miR-301a precursor (Life Technologies) in 12-well plate using Lipofectamine 2000 (Life Technologies) according to manufacturer’s recommendations. After 24hr, Dual-Luciferase Assay (Promega) was performed to measure the reporter activity.

The predicted Fra-2 binding sequences on GLIPR1 and MIR301A promoter were amplified by PCR using specific primers. PCR products were digested with NheI and XhoI ((New England Biolabs) and cloned into pGL3-promoter vector (Promega). The mutants of Fra-2 binding sequence on GLIPR1 and MIR301A promoter were generated using QuikChange II XL Site-Directed Mutagenesis Kit (Agilent), according to the manufacturer’s protocol. H1437 cells were co-transfected with 1 μg of pGL3-promoter constructs and 100 ng of pRL-TK (Renilla luciferase control reporter, Promega) and 150 ng of pCMV6-Entry-Fra2 in 12-well plate using Lipofectamine 2000 (Life Technologies) according to manufacturer’s recommendations. After 24hr, Dual-Luciferase Assay (Promega) was performed to measure the reporter activity.

Primers are listed in supplementary Table 3.

**Bisulfite sequence and 5-Aza-2’-deoxycytidine treatment**

Genomic DNA from cell lines was isolated using DNeasy Blood & Tissue Kit (Qiagen), and bisulfite conversion was performed using EZ DNA Methylation- Kit (ZymoResearch), according to the manufacturer’s instructions. The methylation-specific PCR primers for the amplification of methylated and un-methylated promoter of GLIPR1 was described in (Xiao, J Cancer Res Clin Oncol. 2011). Bisulfite-converted genomic DNA was amplified using Hot start ZymoTaq DNA Polymerase (ZymoResearch). The methylated purified PCR fragments were separated by electrophoresis on a 2% agarose gel with ethidium bromide. PCR fragments were purified and cloned into pGEMT vector (Promega) and five individual clones were sequenced. Primers for sequencing are listed in supplementary Table 3.

H1437, H1299 and H1975 cells were treated with 5-Aza-2’-deoxycytidine (5-Aza-dC; Selleckchem) 1 and 5 μM. The drug was replaced every 24 hr. After 72hr of treatment, cells were collected, and total RNA was extracted.

**MTS cell proliferation assay**

NSCLC cells were transfected and seeded into 96-well culture plate (1500-3000 cells/well, respectively) and after 24hr, were treated with cisplatin and/or T-5224 for 48hr as indicated. Cell viability was assayed using CellTiter 96® AQueous One Solution Cell Proliferation Assay kit (Promega).

**Western blot analysis**

Lung cancer cell lines and lung cancer patients’ samples were lysed in NP40 cell lysis buffer (Invitrogen) supplemented with Protease Inhibitor Cocktail Set III, EDTA-Free (Calbiochem). Lung cancer patients’ samples were fragmented with an electric homogenizer, on ice. Protein concentration was determined by Bradford assay (Bio-Rad), following the manufacturer’s instructions. Protein lysates were separated in 4-20% SDS-PAGE Criterion Precast gel (Bio-Rad) and transferred onto a nitrocellulose membrane (HybondC, Amersham). Membranes were incubated at 4C overnight with anti-Fra-2 (Cell Signaling), anti-GLIPR1 (Santa Cruz Biotechnology), anti-GAPDH (Genetex) and anti-Vinculin (Abcam). After incubation with appropriate horseradish peroxidase-conjugated secondary antibodies (GE Healthcare), signal was detected using Immobilon Forte HPR detection reagent (Millipore). Densitometry plots and signal intensity quantification were obtained using ImageJ software (U. S. National Institutes of Health, Bethesda, Maryland, USA).

**RNA isolation and quantitative Real-time PCR**

Total RNA was isolated from cell lines and human samples using TRIzol (Invitrogen), following the provided instructions. For qRT-PCR, TaqMan miRNA assays from ThermoFisher (miR-301a#000528) were used to detect mature miRNAs. RNU44 (ThermoFisher TaqMan Assay #00194) was used as normalizers. TaqMan gene expression assays from ThermoFisher were used to detect mRNA expression of FOSL2 (Hs01050117_m1) and GLIPR1 (Hs61564146_g1). GAPDH (Hs02786624_g1) was used as normalizers.

**Affymetrix microarray**

A549 cell lines were transfected with pCMV6-Entry-Fra2 (Myc-DDK-tagged) and pCMV6-Entry as control (ORIGENE, Rockville, MD) and, after 48 hours, RNA was collected and isolated with Trizol and RNA clean-up and concentration kit Norgen. Total RNA was treated with RNase-free DNase I Kit by Norgen, in order to avoid DNA contamination. Transcriptome analysis was conducted on A549 samples obtained from three different transfection experiments and was performed with Clariom™ S Assay, Human (Thermo Fisher Scientific).

**Bioinformatics analyses**

Genes (fragments per kilobase of exon per million mapped fragments - FPKM) and miRNA isoforms (reads per million mapped reads or counts per million mapped reads - RPM) L3 expression data, along with patients' clinical data, were downloaded from the Genomic Data Commons Data Portal (https://portal.gdc.cancer.gov/). In this work, we considered a cumulative expression for hsa-miR-301a, generated by the contribution of all miRNA isoforms having up to one nucleotide added/trimmed at both 5'- and 3'-end.

The analysis for Clariom™ S Human Affymetrix panel of ~20K genes was carried out by Transcriptome Analysis Console software (v4 - Thermo Fisher Scientific) and it consists of three main steps: 1) data quality control; 2) normalization (signal space transformation robust multiple-array average); 3) differentially expression analysis employing the eBayes method from limma R package.

**Statistical analysis**

All graphs and statistical analyses were performed using PRISM (version 9, GraphPad, Inc.). In all experiments, differences were considered statistically significant when p-value was <0.05. Statistical analyses included paired and unpaired t-tests, Mann Whitney un-paired t-test, Spearman correlation test, non-parametric Wilcoxon test, used as appropriate and as specified in each figure.

**SUPPLEMENTARY REFERENCE**

1. Thai, A. A., Solomon, B. J., Sequist, L. V., Gainor, J. F. & Heist, R. S. Lung cancer. *The Lancet* **398**, 535–554 (2021).

2. Arbour, K. C. & Riely, G. J. Systemic Therapy for Locally Advanced and Metastatic Non–Small Cell Lung Cancer: A Review. *JAMA* **322**, 764–774 (2019).

3. Majeed, U., Manochakian, R., Zhao, Y. & Lou, Y. Targeted therapy in advanced non-small cell lung cancer: current advances and future trends. *J. Hematol. Oncol.J Hematol Oncol* **14**, 108 (2021).

4. Scarpino, S. *et al.* High prevalence of ALK+/ROS1+ cases in pulmonary adenocarcinoma of adoloscents and young adults. *Lung Cancer Amst. Neth.* **97**, 95–98 (2016).

5. Gonzalez-Rajal, A., Hastings, J. F., Watkins, D. N., Croucher, D. R. & Burgess, A. Breathing New Life into the Mechanisms of Platinum Resistance in Lung Adenocarcinoma. *Front. Cell Dev. Biol.* **8**, (2020).

6. Zheng, J.-Z. *et al.* Elevated miR-301a expression indicates a poor prognosis for breast cancer patients. *Sci. Rep.* **8**, 2225 (2018).

7. Nam, R. K. *et al.* MiR-301a regulates E-cadherin expression and is predictive of prostate cancer recurrence. *The Prostate* **76**, 869–884 (2016).

8. Yue, X., Lan, F. & Xia, T. Hypoxic Glioma Cell-Secreted Exosomal miR-301a Activates Wnt/β-catenin Signaling and Promotes Radiation Resistance by Targeting TCEAL7. *Mol. Ther.* **27**, 1939–1949 (2019).

9. Wang, X. *et al.* Hypoxic Tumor-Derived Exosomal miR-301a Mediates M2 Macrophage Polarization via PTEN/PI3Kγ to Promote Pancreatic Cancer Metastasis. *Cancer Res.* **78**, 4586–4598 (2018).

10. Lu, Z. *et al.* miR-301a as an NF-κB activator in pancreatic cancer cells. *EMBO J.* **30**, 57–67 (2011).

11. Li, X. *et al.* miR-301a promotes lung tumorigenesis by suppressing Runx3. *Mol. Cancer* **18**, 99 (2019).

12. Cao, G. *et al.* Intronic miR-301 feedback regulates its host gene, ska2, in A549 cells by targeting MEOX2 to affect ERK/CREB pathways. *Biochem. Biophys. Res. Commun.* **396**, 978–982 (2010).

13. Ma, X. *et al.* Modulation of tumorigenesis by the pro-inflammatory microRNA miR-301a in mouse models of lung cancer and colorectal cancer. *Cell Discov.* **1**, 15005 (2015).

14. Guo, J. *et al.* miR-301a-3p induced by endoplasmic reticulum stress mediates the occurrence and transmission of trastuzumab resistance in HER2-positive gastric cancer. *Cell Death Dis.* **12**, 1–14 (2021).

15. Salim, H. M. *et al.* Abstract 163: mRNA and miRNA profiling of platinum-resistant non-small cell lung cancer cell clones identifies potential targets involved in DNA metabolism and DNA replication. *Cancer Res.* **71**, 163 (2011).

16. Wang, J. *et al.* miR-301a Suppression within Fibroblasts Limits the Progression of Fibrosis through the TSC1/mTOR Pathway. *Mol. Ther. - Nucleic Acids* **21**, 217–228 (2020).

17. Huang, Y. *et al.* MiR-301a-5p/SCIN promotes gastric cancer progression via regulating STAT3 and NF-κB signaling. *J. Cancer* **12**, 5394–5403 (2021).

18. Mycko, M. P. *et al.* microRNA-301a regulation of a T-helper 17 immune response controls autoimmune demyelination. *Proc. Natl. Acad. Sci.* **109**, E1248–E1257 (2012).

19. Arnold, S. *et al.* Fra-2 overexpression upregulates pro-metastatic cell-adhesion molecules, promotes pulmonary metastasis, and reduces survival in a spontaneous xenograft model of human breast cancer. *J. Cancer Res. Clin. Oncol.* (2021) doi:10.1007/s00432-021-03812-2.

20. Chen, G.-L. *et al.* Fra-2/AP-1 regulates melanoma cell metastasis by downregulating Fam212b. *Cell Death Differ.* **28**, 1364–1378 (2021).

21. Yin, J. *et al.* HGF/MET Regulated Epithelial-Mesenchymal Transitions And Metastasis By FOSL2 In Non-Small Cell Lung Cancer. *OncoTargets Ther.* **12**, 9227–9237 (2019).

22. Sarode, P. *et al.* Reprogramming of tumor-associated macrophages by targeting β-catenin/FOSL2/ARID5A signaling: A potential treatment of lung cancer. *Sci. Adv.* **6**, eaaz6105.

23. Alfraidi, A. *et al.* Dissecting the roles of Fra proteins in lung adenocarcinoma. *Ann. Oncol.* **27**, vi1 (2016).

24. Zhang, Q., Adiseshaiah, P. & Reddy, S. P. Matrix Metalloproteinase/Epidermal Growth Factor Receptor/Mitogen-Activated Protein Kinase Signaling Regulate fra-1 Induction by Cigarette Smoke in Lung Epithelial Cells. *Am. J. Respir. Cell Mol. Biol.* **32**, 72–81 (2005).

25. Karamouzis, M. V., Konstantinopoulos, P. A. & Papavassiliou, A. G. The Activator Protein-1 Transcription Factor in Respiratory Epithelium Carcinogenesis. *Mol. Cancer Res.* **5**, 109–120 (2007).

26. Li, Z. *et al.* lncRNA UCA1 Mediates Resistance to Cisplatin by Regulating the miR-143/FOSL2-Signaling Pathway in Ovarian Cancer. *Mol. Ther. Nucleic Acids* **17**, 92–101 (2019).

27. Chen, X. *et al.* lncRNA UCA1 Promotes Gefitinib Resistance as a ceRNA to Target FOSL2 by Sponging miR-143 in Non-small Cell Lung Cancer. *Mol. Ther. Nucleic Acids* **19**, 643–653 (2020).

28. Hossian, A. K. M. N. *et al.* Advanced bioinformatic analysis and pathway prediction of NSCLC cells upon cisplatin resistance. *Sci. Rep.* **11**, 6520 (2021).

29. Gong, X. *et al.* GLIPR1 modulates the response of cisplatin-resistant human lung cancer cells to cisplatin. *PLOS ONE* **12**, e0182410 (2017).

30. Wang, J. *et al.* Glioma pathogenesis-related protein 1 performs dual functions in tumor cells. *Cancer Gene Ther.* 1–11 (2021) doi:10.1038/s41417-021-00321-9.

31. Agarwal, V., Bell, G. W., Nam, J.-W. & Bartel, D. P. Predicting effective microRNA target sites in mammalian mRNAs. *eLife* **4**, e05005 (2015).

32. Sheng, X., Bowen, N. & Wang, Z. GLI pathogenesis-related 1 functions as a tumor-suppressor in lung cancer. *Mol. Cancer* **15**, 25 (2016).

33. Gazon, H., Barbeau, B., Mesnard, J.-M. & Peloponese, J.-M. Hijacking of the AP-1 Signaling Pathway during Development of ATL. *Front. Microbiol.* **8**, (2018).

34. Rouillard, A. D. *et al.* The harmonizome: a collection of processed datasets gathered to serve and mine knowledge about genes and proteins. *Database J. Biol. Databases Curation* **2016**, baw100 (2016).

35. Luo, Y. *et al.* New developments on the Encyclopedia of DNA Elements (ENCODE) data portal. *Nucleic Acids Res.* **48**, D882–D889 (2020).

36. Ren, C. *et al.* RTVP-1, a tumor suppressor inactivated by methylation in prostate cancer. *Cancer Res.* **64**, 969–976 (2004).

37. Xiao, Y.-H. *et al.* Identification of GLIPR1 tumor suppressor as methylation-silenced gene in acute myeloid leukemia by microarray analysis. *J. Cancer Res. Clin. Oncol.* **137**, 1831 (2011).

38. Makino, H. *et al.* A selective inhibition of c-Fos/activator protein-1 as a potential therapeutic target for intervertebral disc degeneration and associated pain. *Sci. Rep.* **7**, 16983 (2017).

39. Yao, C. D. *et al.* AP-1 and TGFß cooperativity drives non-canonical Hedgehog signaling in resistant basal cell carcinoma. *Nat. Commun.* **11**, 5079 (2020).

40. Pandey, K. *et al.* Molecular mechanisms of resistance to CDK4/6 inhibitors in breast cancer: A review. *Int. J. Cancer* **145**, 1179–1188 (2019).

41. Rampioni Vinciguerra, G. L. *et al.* CDK4/6 Inhibitors in Combination Therapies: Better in Company Than Alone: A Mini Review. *Front. Oncol.* **12**, 891580 (2022).

42. Luo, G. *et al.* miR-301a plays a pivotal role in hypoxia-induced gemcitabine resistance in pancreatic cancer. *Exp. Cell Res.* **369**, 120–128 (2018).

43. Rosenzweig, T. *et al.* Related to testes-specific, vespid, and pathogenesis protein-1 (RTVP-1) is overexpressed in gliomas and regulates the growth, survival, and invasion of glioma cells. *Cancer Res.* **66**, 4139–4148 (2006).

44. Preston, G. A. *et al.* Induction of apoptosis by c-Fos protein. *Mol. Cell. Biol.* **16**, 211–218 (1996).

45. Bossy-Wetzel, E., Bakiri, L. & Yaniv, M. Induction of apoptosis by the transcription factor c-Jun. *EMBO J.* **16**, 1695–1709 (1997).

46. Mahner, S. *et al.* C-Fos expression is a molecular predictor of progression and survival in epithelial ovarian carcinoma. *Br. J. Cancer* **99**, 1269–1275 (2008).

Figure. S1.

**
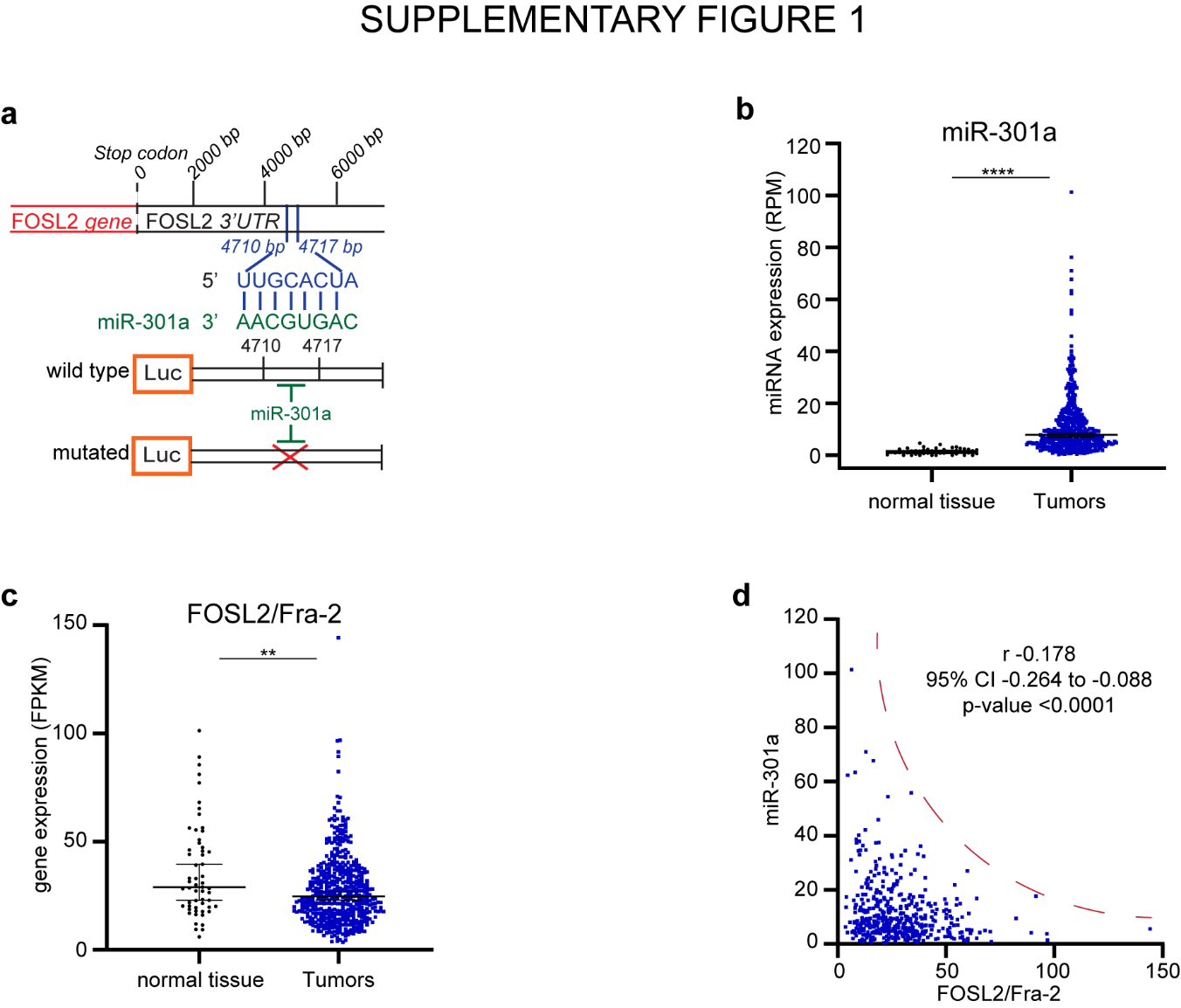
**

**Supplementary Figure 1. miR-301a expression in adenocarcinomas from TCGA database.** **a.** Schematic representation of miR-301a binding site on FOSL2/Fra-2 3’UTR and its deletion. **b**, **c**. Box plots showing miR-301a (b) and FOSL2/Fra-2 (c) expression in lung adenocarcinoma samples (n=507 for miR-301a, n=494 for FOSL2/Fra-2) versus normal tissues (n=46 for miR-301a, n=57 for FOSL2/Fra-2) from TCGA database. Mann-Whitney test was used for statistical analysis. RPM stands for reads per million; FPKM stands for fragments per kilobases per million. **, 0.001< p-value ≤0.01; ****, p-value ≤0.0001. **d.** Scatter plot showing the negative correlation between the expression of miR-301a and FOSL2/Fra-2 in TCGA cohort of lung adenocarcinoma samples (n=494). Spearman correlation test was used for statistical analysis.

Figure. S2.

**
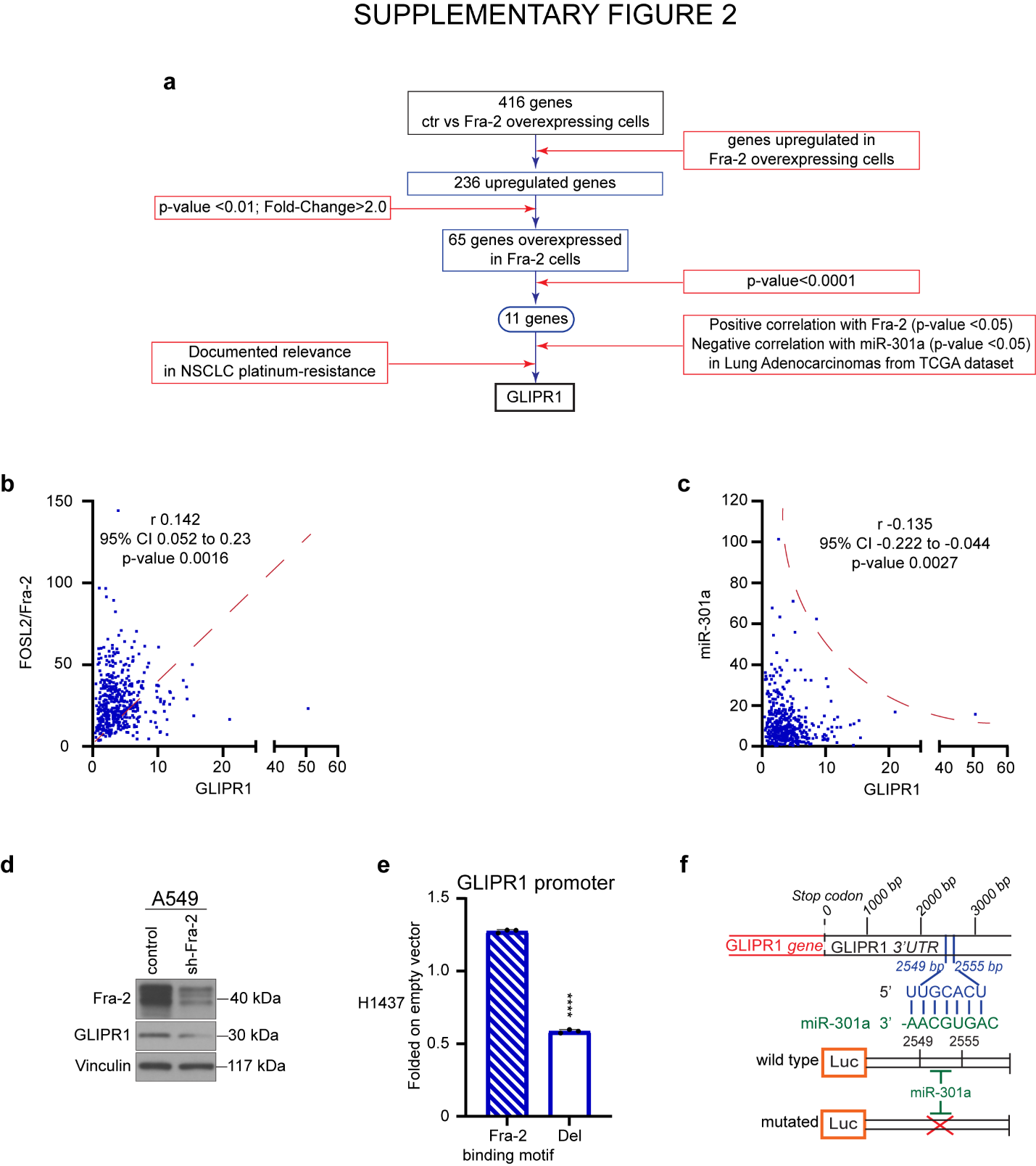
**

**Supplementary Figure 2. GLIPR1 as a direct and indirect target of miR-301a via Fra-2 transcriptional activity.** **a.** Flow chart of the inclusion criteria for gene selection after microarray analysis on A549 cells overexpressing or not Fra-2. **b, c.** Scatter plots showing the correlation between FOSL2/Fra-2 and GLIPR1 (b) and the negative correlation between miR-301a and GLIPR1 (c) in TCGA cohort of lung adenocarcinoma samples (n=494). Spearman correlation test was used for statistical analysis. **d.** Western blot analysis of Fra-2 and GLIPR1 in A549 cells silenced or not for Fra-2, as indicated. Vinculin was used as loading control. **e.** Histogram reporting the normalized luciferase activity of GLIPR1 and GLIPR1 mutated promoter, containing a deletion of Fra-2 binding sequence in H1437 cells. Reported results were folded on the empty vector and unpaired t-test was used for statistical analysis. **** p-value <0.0001. **f.** Schematic representation of miR-301a binding site on GLIPR1 3’UTR and its deletion.

Figure. S3.

**
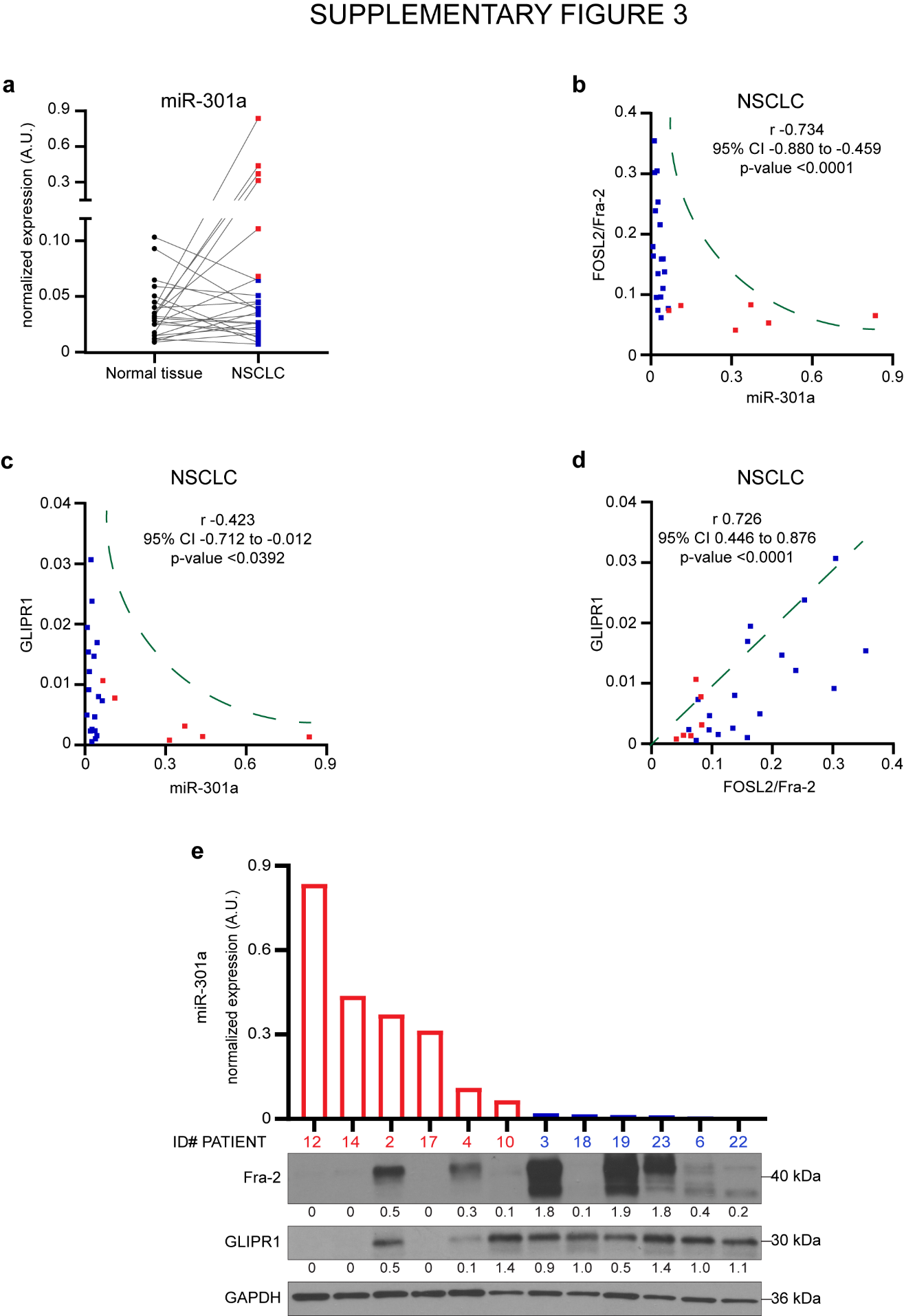
**

**Supplementary Figure 3. MiR-301a and its targets expression in an independent cohort of NSCLC patients. a**. Bipartite graph showing miR-301a expression in an internal cohort of 24 paired NSCLC patients versus their normal counterpart. Red squares represent the patients’ samples with high miR-301a expression. **b-d.** Scatter plots representing the anticorrelation between miR-301a and FOSL2/Fra-2 (b), miR-301a and GLIPR1 (c) and the correlation between FOSL2/Fra-2 and GLIPR1 (d) in the internal cohort of 24 NSCLC samples. Spearman correlation test was used for statistical analysis. **e.** Western blot analysis of Fra-2 and GLIPR1 in NSCLC patient’ lysates based on miR-301a expression levels, represented in the histogram. Patients with high miR-301a expression (high quartile) are indicated in red and patients with low or undetectable miR-301a expression (low quartile) are indicated in blue. For qRT-PCR, RNU44 was used as normalizer. Numbers indicate relative quantification of Fra-2 and GLIPR1 expression respect to GAPDH loading control.

Figure. S4.


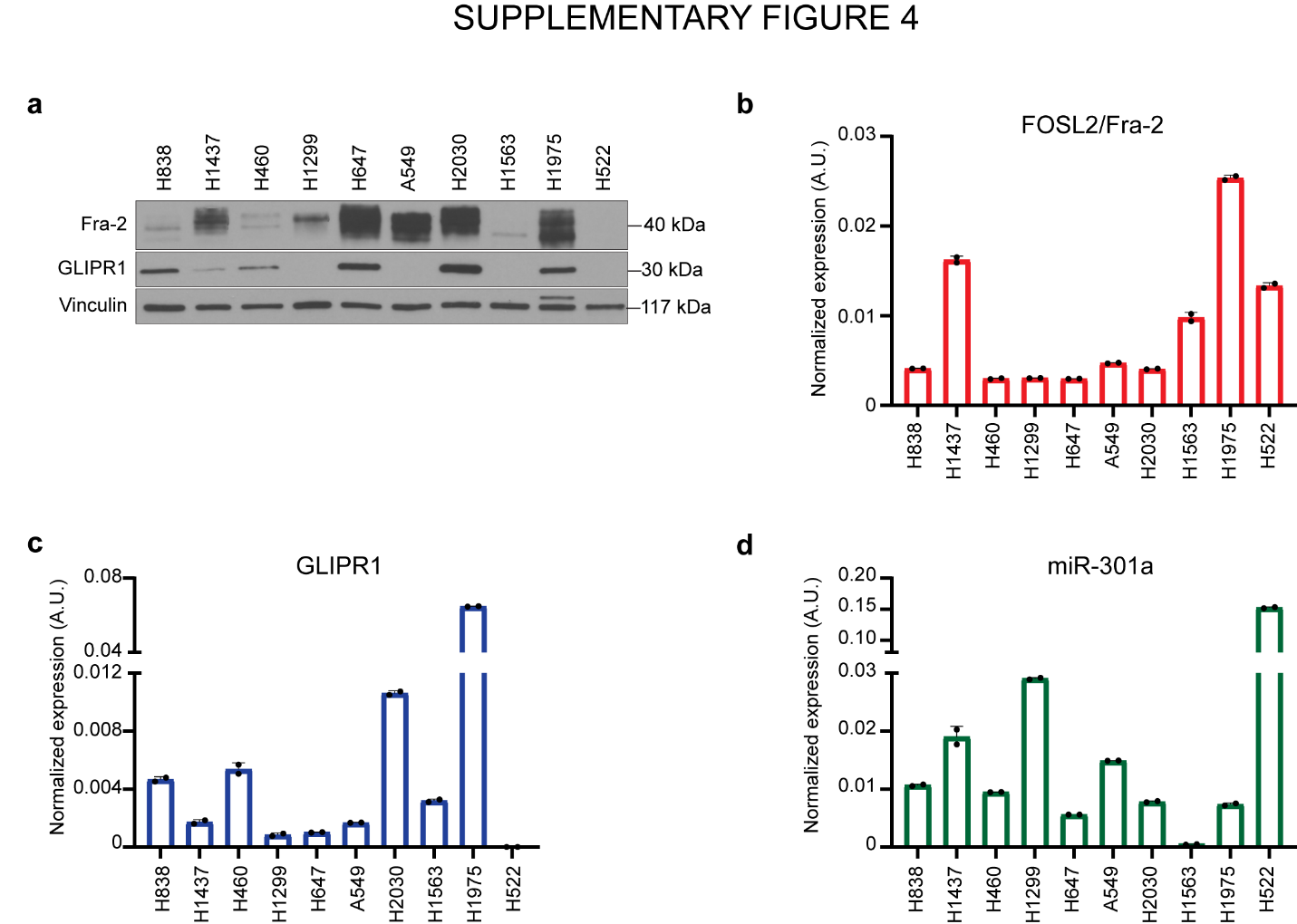


**Supplementary Figure 4. Expression of miR-301a targets in NSCLC cell lines.** **a.** Western blot analysis of Fra-2 and GLIPR1 in different NSCLC cell lines, as indicated. Vinculin was used as loading control. **b-d.** Histograms representing FOSL2/Fra-2 (b), GLIPR1 (c) and miR-301a (d) expression by qRT-PCR in different NSCLC cell lines. GAPDH was used as normalizer for genes. RNU44 was used as normalizer for miRNA.

Figure. S5.


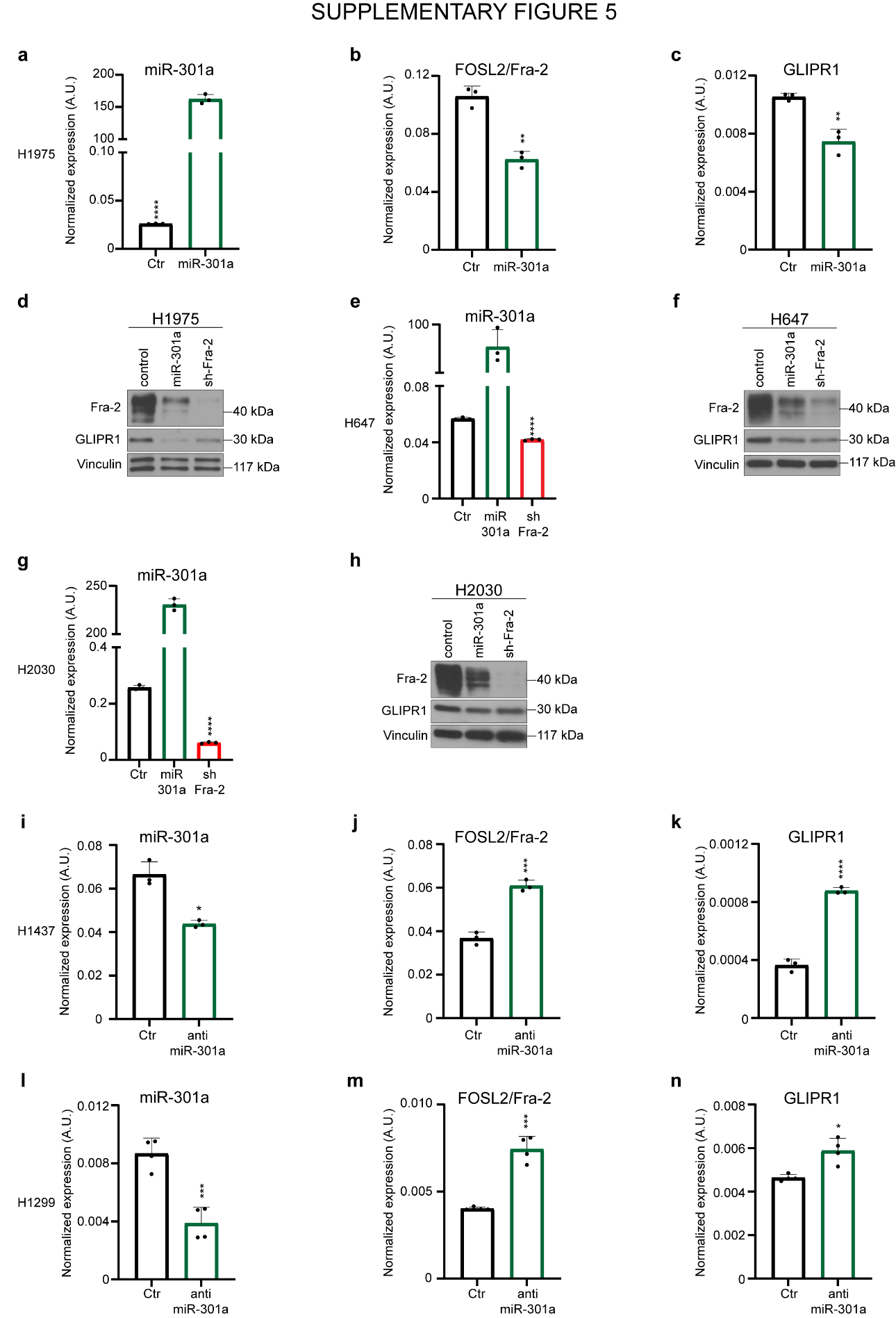


**Supplementary Figure 5.** **Effects of miR-310a modulation on Fra-2/GLIPR1 expression in NSCLC cell lines.** **a-c.** miR-301a (a), FOSL2/Fra-2 (b) and GLIPR1 (c) expression by qRT-PCR after miR-301a overexpression in H1975 cell line. **d.** Western blot analysis of Fra-2 and GLIPR1 in miR-301a overexpressing and FOSL2/Fra-2 silenced H1975 cell line. **e, f.** miR-301a expression by qRT-PCR (e) and western blot analysis of Fra-2 and GLIPR1 (f) in miR-301a overexpressing and FOSL2/Fra-2 silenced H647 cell line. Statistical significance of miR-301a expression in FOSL2/Fra-2 silenced cells was evaluated compared to the control. **g, h.** miR-301a expression by qRT-PCR (g) and western blot analysis of Fra-2 and GLIPR1 (h) in miR-301a overexpressing and FOSL2/Fra-2 silenced H2030 cell line. Statistical significance of miR-301a expression in FOSL2/Fra-2 silenced cells was evaluated compared to the control. **i-k**. miR-301a (i), FOSL2/Fra-2 (j) and GLIPR1 (k) expression after miR-301a silencing in H1437 cell line by qRT-PCR. **l-n**. miR-301a (l), FOSL2/Fra-2 (m) and GLIPR1 (n) expression after miR-301a silencing in H1299 cell line by qRT-PCR. For qRT-PCR analysis, GAPDH was used as normalizer for genes and RNU44 for miRNA. For Western blot analysis, vinculin was used as loading control. * p-value≤0.05, ** p-value ≤0.01, *** p-value <0.001, **** p-value <0.0001.

Figure. S6.


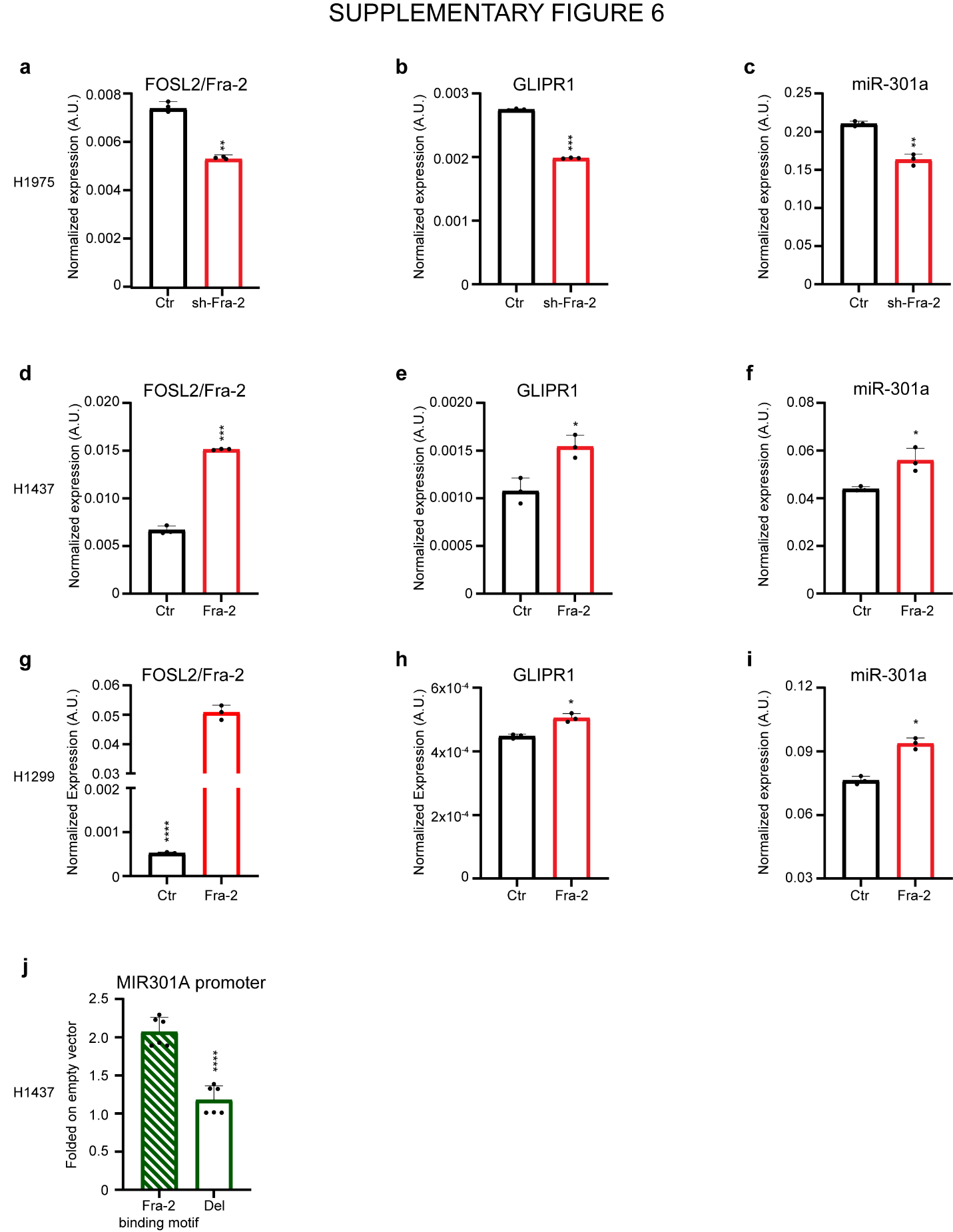


**Supplementary Figure 6.** **Effects of Fra-2 modulation on miR-301a/GLIPR1 expression in different NSCLC lines.** **a-c**. FOSL2/Fra-2 (a), GLIPR1 (b) and miR-301a (c) expression by qRT-PCR in Fra-2 silenced H1975 cell line. **d-i.** FOSL2/Fra-2 (d, g), GLIPR1 (e, h) and miR-301a (f, i) expression by qRT-PCR in Fra-2 overexpressing H1437 (d-f) and H1299 (g-i) cell lines, respectively. For qRT-PCR analysis, GAPDH was used as normalizer for genes and RNU44 for miRNA. **j.** Histogram reporting the normalized luciferase activity of MIR301A and MIR301A mutated promoter, containing a deletion of Fra-2 binding sequence in H1437 cells. Reported results were folded on the empty vector and unpaired t-test was used for statistical analysis. * p-value≤0.05, ** p-value ≤0.01, *** p-value <0.001, **** p-value <0.0001.

Figure. S7.


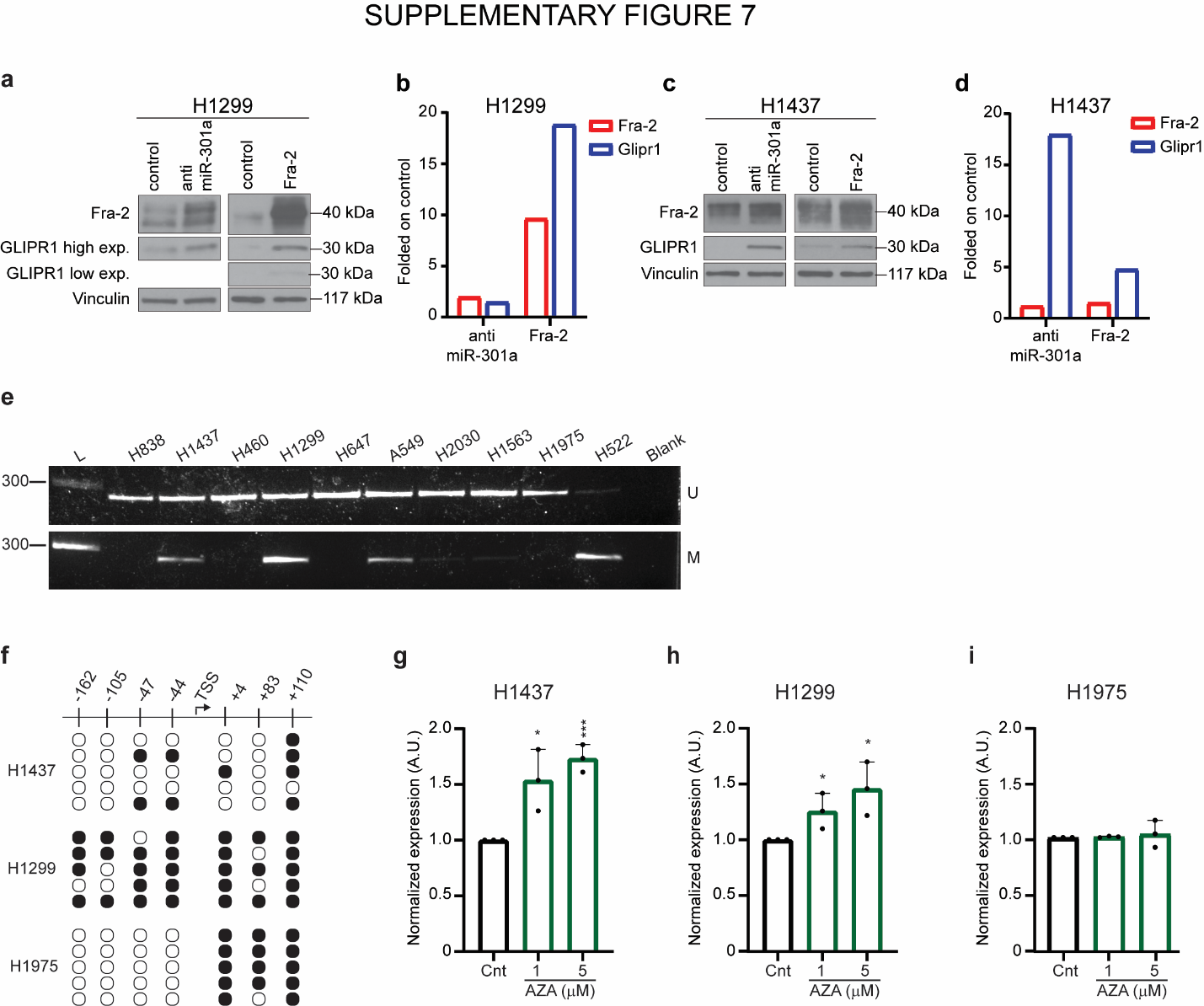


**Supplementary Figure 7. Methylation status of GLIPR1 promoter in NSCLC cell lines.** **a-d.** Western blot analysis of Fra-2 and GLIPR1 and their relative quantification in miR-301a-silenced and FOSL2-overexpressed H1299 (a, b) and H1437 (c, d) cell lines. Vinculin was used as loading control. Histograms represent Fra-2 and GLIPR1 quantification folded on control in the indicated samples. **e.** Methylation specific PCR (MSP) analysis of GLIPR1 promoter in bisulfite-converted DNA from 10 NSCLC lines. U, unmethylated gene; M, methylated gene; L, 1Kb DNA Marker. **f.** Bisulfite sequencing analysis of GLIPR1 promoter. The CpG sites is spanning from -162 to +110 nucleotides where 0 is the transcription start site (TSS) of GLIPR1. Each vertical bar represents a single CpG. Black circles indicate methylated CpG sites and white circles represent unmethylated CpG sites. **g-i**. Expression of GLIPR1 in untreated (Cnt) or treated with two different concentrations of DNA demethylating agent 5-aza-2’deoxycytidine H1437 (g), H1299 (h) and H1975 (i) cell lines. * p-value≤0.05, *** p-value <0.001.

Figure. S8.

**
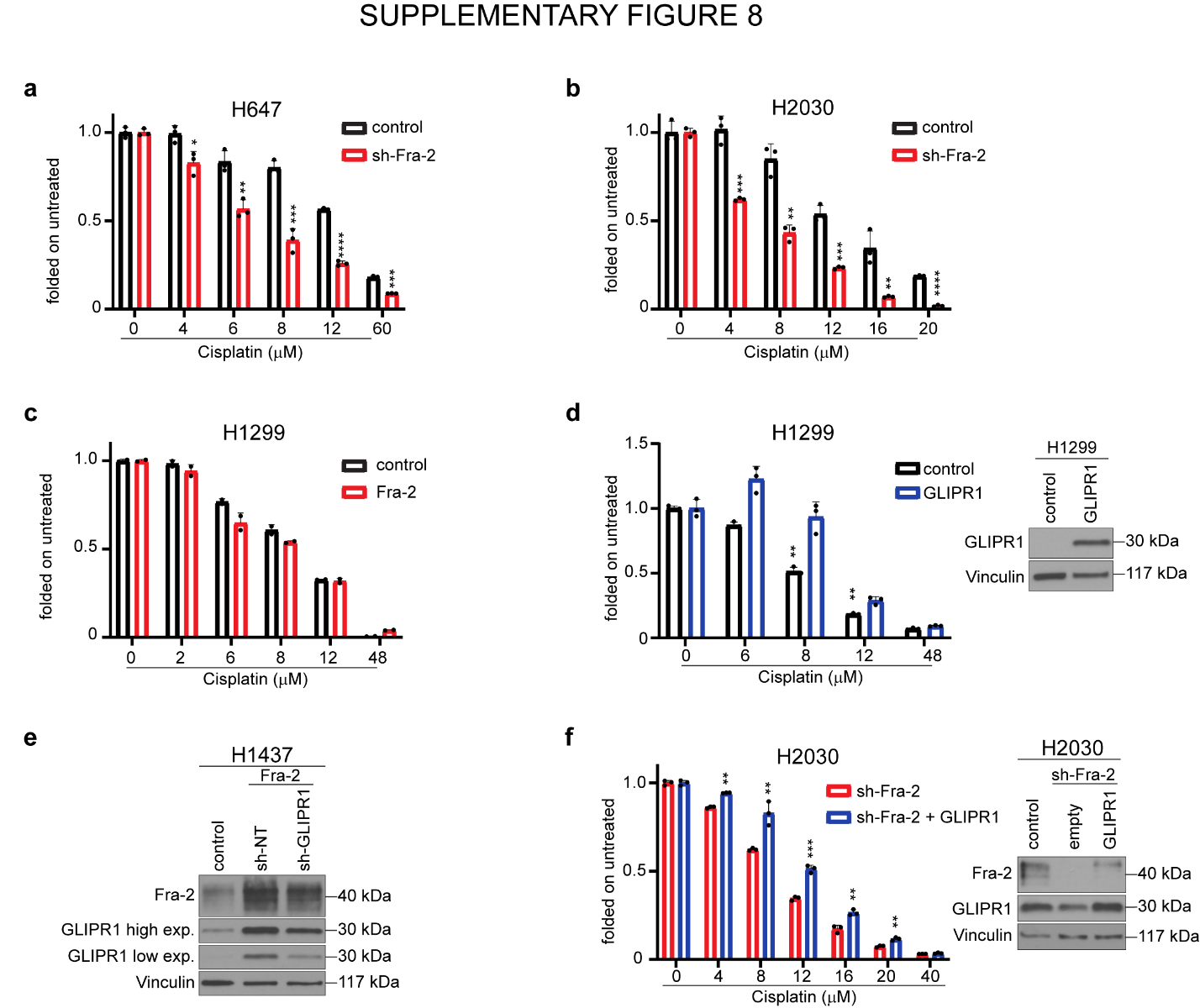
**

**Supplementary Figure 8. Fra-2 expression affects cisplatin sensitivity via GLIPR1. a-c.** MTS cell viability assay of FOSL2/Fra-2 silenced H647 (a) and H2030 (b) and Fra-2 overexpressing H1299 (c) cells treated with increasing concentrations of cisplatin. Data are collected after 48hr of cisplatin treatment and are folded over the untreated cells. **d.** On the left, viability assay of control and GLIPR1 overexpressing H1299 cells treated with increasing concentrations of cisplatin. On the right, western blot analysis of the indicated proteins in H1299 cells used in viability assay. Vinculin was used as loading control. **e.** Western blot analysis of Fra-2 and GLIPR1 in control and Fra-2 overexpressing H1437 cells, silenced or not for GLIPR1. Vinculin was used as loading control. **f.** On the left, MTS cell viability assay of FOSL2/Fra-2 silenced and overexpressing or not GLIPR1 H2030 cells treated with increasing concentrations of cisplatin. Data are collected after 48hr of cisplatin treatment and are folded over the untreated cells. On the right, western blot analysis of indicated proteins in H2030 cells, used in viability assay. Vinculin was used as loading control. * p-value≤0.05, ** p-value ≤0.01, *** p-value <0.001, **** p-value <0.0001.

Figure. S9.


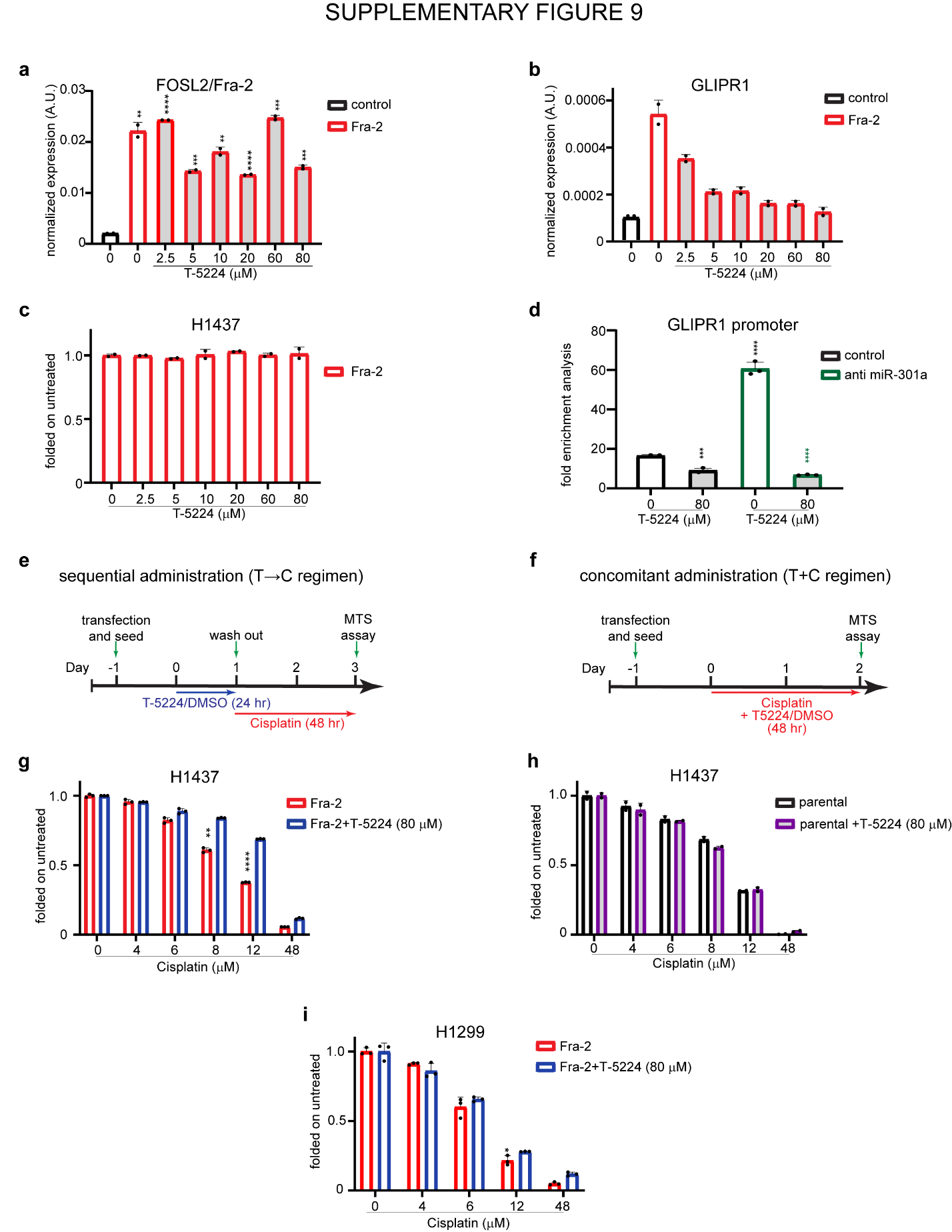


**Supplementary Figure 9.** **Fra-2 inhibition affects cisplatin sensitivity via GLIPR1.** **a, b.** FOSL2/Fra-2 (a) and GLIPR1 (b) expression by qRT-PCR in FOSL2/Fra-2-overexpressing H1437 cell line treated with increasing doses of T-5224. Statistical significance was evaluated comparing FOSL2/Fra-2 levels in Fra-2 overexpressing cells compared to control. **c.** Proliferation assay of FOSL2/Fra-2-overexpressing H1437 cells treated with increasing doses of T-5224. **d.** Histogram showing the binding of anti-Fra2 antibody to the GLIPR1 promoter by Chromatin immunoprecipitation (ChiP) assay in control and miR-301a-silenced H1437 cells treated or not with 80 μM T-5224. Data represent the fold enrichment over the IgG control. Black asterixis indicate the statistical significance over the untreated control, while green asterixis over the untreated miR-301a silenced H1437 cells. **e.** Outline of sequential treatment (T→C regimen): T-5224 (80 μM) for 24hr, wash out and increasing concentrations of cisplatin for 48hr. **f, g.** Outline of concomitant treatment (T+C regimen): T-5224 (80 μM) and increasing concentrations of cisplatin for 48hr (f). MTS cell viability assay of Fra-2-overexpressing H1437 cells treated with concomitant schedule (g). **h.** Proliferation assay of H1437 parental cells treated or not with sequential administration of T-5224 (80 μM) and increasing doses of cisplatin (T→C regimen). **i.** MTS cell viability assay of Fra-2 overexpressing H1299 cells treated or not with sequential administration of T-5224 (80 μM) and increasing doses of cisplatin. Statistical significance was evaluated by Student’s t-test and asterisks mark the statistically differences as follow: * p-value≤0.05, ** p-value ≤0.01, *** p-value <0.001, **** p-value <0.0001.

**Supplementary Table 1. List of differentially expressed genes in A549 cells transfected or not with Fra-2**

| **Supplementary Table 2. Lung Cancer Cohort** | |
| --- | --- |
| **Features** | **Value** |
| **Age at diagnosis** |  |
| average | 64 |
| range | 50-76 |
| **Gender** | **n. (%)** |
| Female | 15 (62.5%) |
| Male | 9 (37.5%) |
| **Histotype** | **n. (%)** |
| Adenocarcinoma | 10 (42%) |
| Squamous cell carcinoma | 8 (33.5%) |
| adenosquamous | 1 (4%) |
| carcinoid | 3 (12.5%) |
| not specified NSCLC | 2 (8%) |
| **Tumor Grade** | **n. (%)** |
| G1 | 4 (16.5%) |
| G2 | 8 (33.5%) |
| G3 | 9 (37.5%) |
| not evaluated | 3 (12.5%) |
| **TNM Staging** | **n. (%)** |
| T1 | 7 (29%) |
| T2 | 9 (37.5%) |
| T3 | 8 (33.5%) |

**Supplementary Table 3. List of Primers used in this study**

| Name of primer | Sequence 5’-3’ |
| --- | --- |
| psiCHECK2 FOSL2 Fw | GAAATGGTCCCATTGGAGAGT |
| psiCHECK2 FOSL2 Rv | GCTACTCAACTGAAAGTGGAAATG |
| psiCHECK2 GLIPR1 Fw | GACCTGTCAGCATCCTTTAGTC |
| psiCHECK2 GLIPR1 Rv | CAAGCAGAAGCCATCAGTAAGA |
| Bisulfite seq GLIPR1 seg1 Fw | TGAAAATTATTGAAAAGATAGGGTTAAG |
| Bisulfite seq GLIPR1 seg1 Rv | CCTTAAAAAACTACAATCCAAAACC |
| Bisulfite seq GLIPR1 seg2 Fw | AAGGTTTTATGTTAGATAAAGTATG |
| Bisulfite seq GLIPR1 seg2 Rv | CTAACTATTAATTTCACCTCTAATC |
| chIP GLIPR1 promoter Fw | GGATTACGCAAGCAACCA |
| chIP GLIPR1 promoter Rev | GCCTGCTTCTCATCTTCTTC |
| chIP MIR301A promoter Fw | AGCTCCCTAAAGTCTCATCC |
| chIP MIR301A promoter Rev | ATTGTCTGGCATGCTGTTAG |
| pGL3 GLIPR1 promoter Fw | GCTAACCTGGTCTGCACAATA |
| pGL3 GLIPR1 promoter Rev | GCCACACACACACAAATAACC |
| pGL3 MIR301A promoter Fw | TGTAGCAGCAACGGAAATGA |
| pGL3 MIR301A promoter Fw | CGGACTCCACCCAAATTTACT |
