# Supplementary material for: Role of the miR-301a/Fra-2/GLIPR1 axis in lung cancer cisplatin resistance
Source: Signal Transduct Target Ther. 2023 Jan 27;8:37. doi: 10.1038/s41392-022-01228-z (PMC9879967; doi:10.1038/s41392-022-01228-z)
Supplement: Supplementary file 3 — original and uncropped films of western Blot [file 41392_2022_1228_MOESM3_ESM.pptx]

## Slide 1
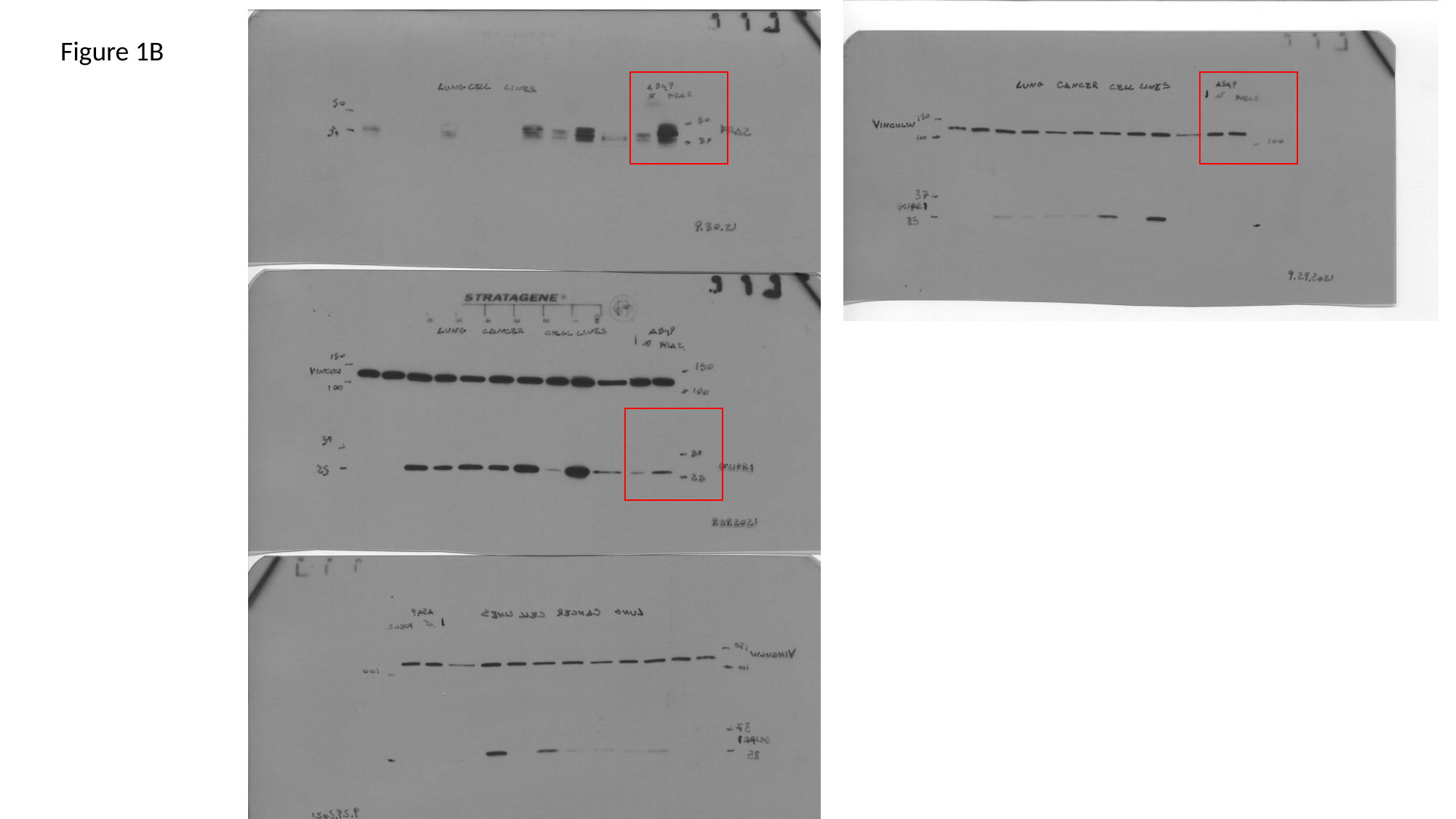

Figure 1B

## Slide 2
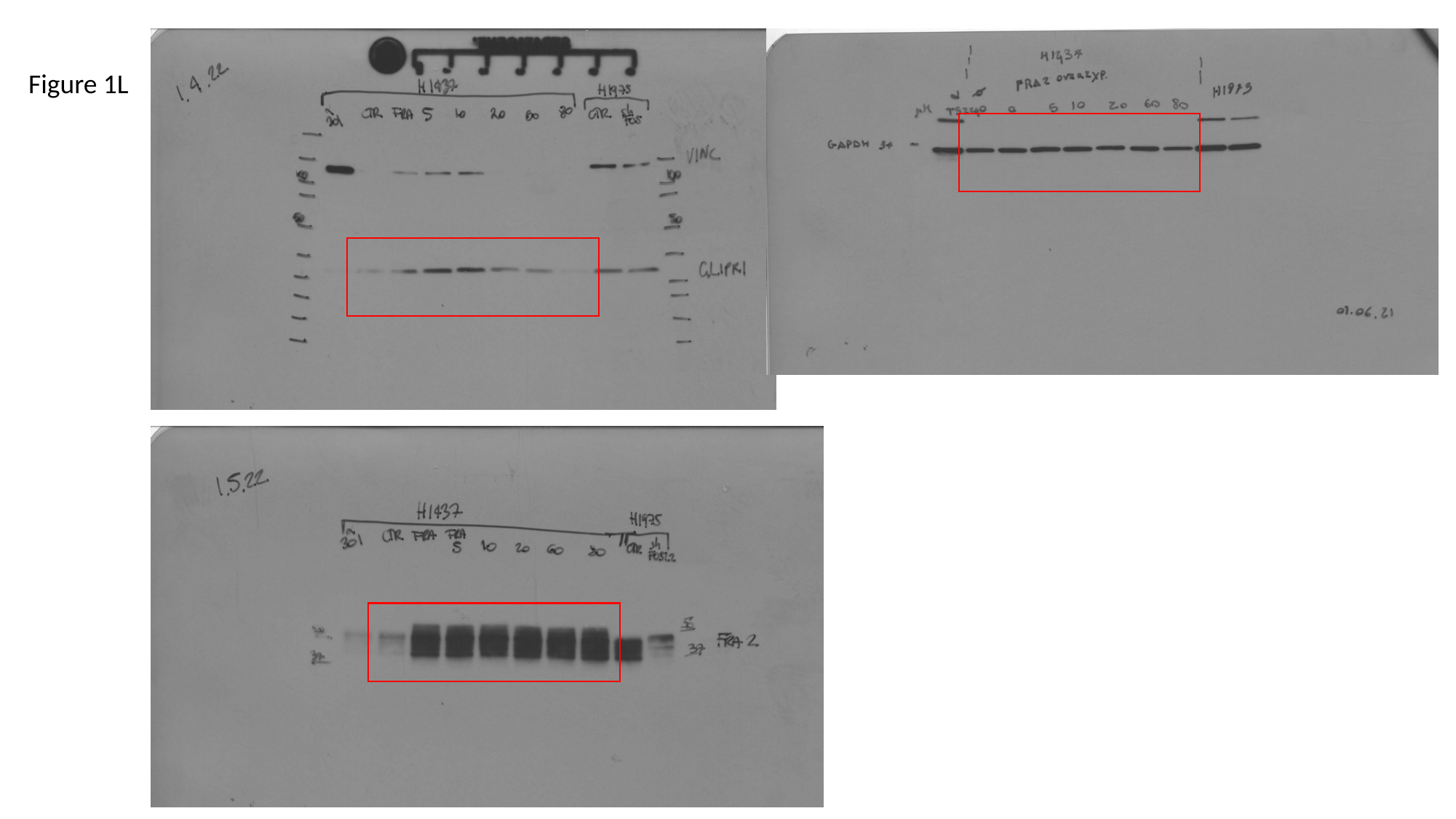

Figure 1L

## Slide 3
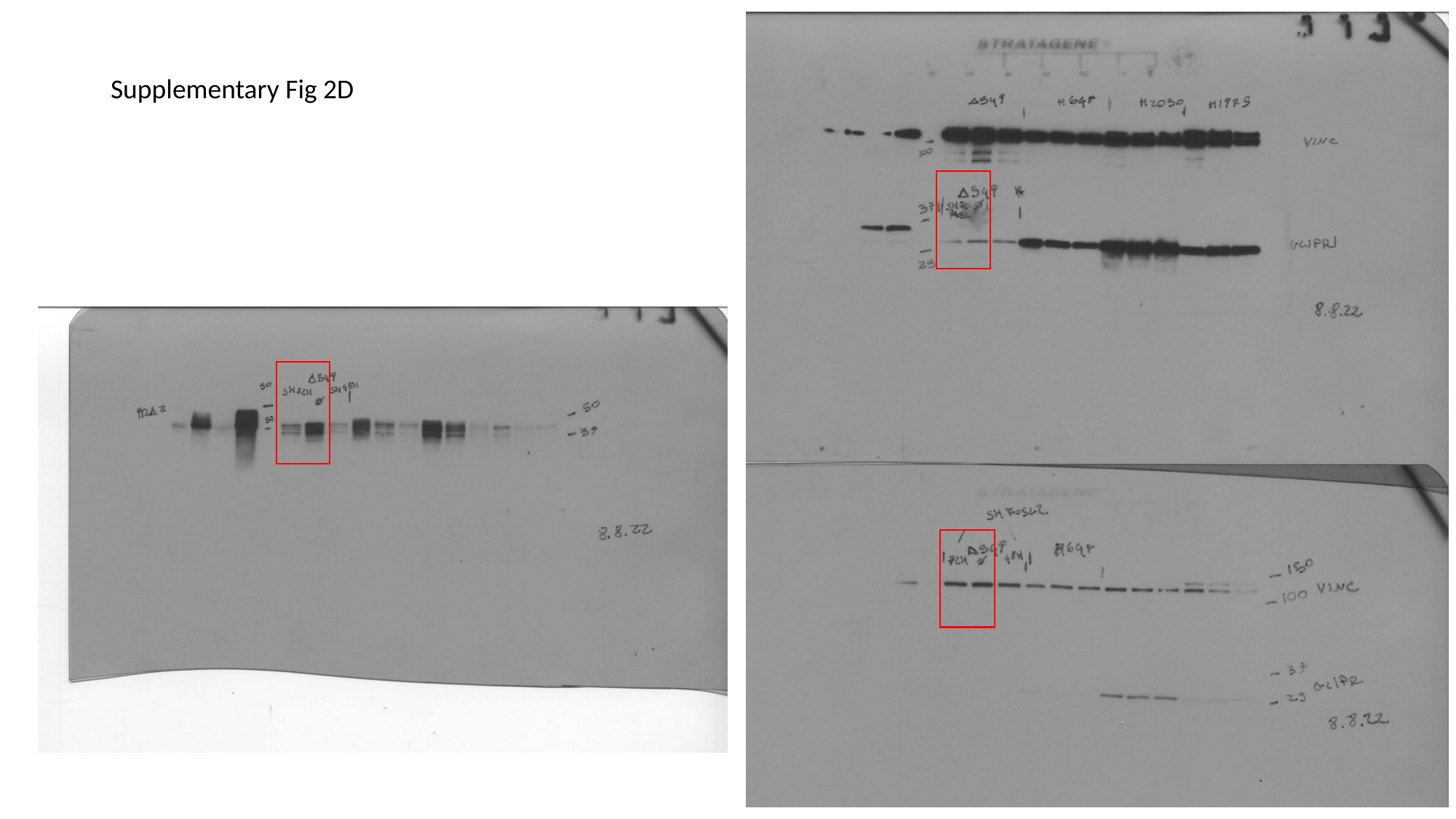

Supplementary Fig 2D

## Slide 4
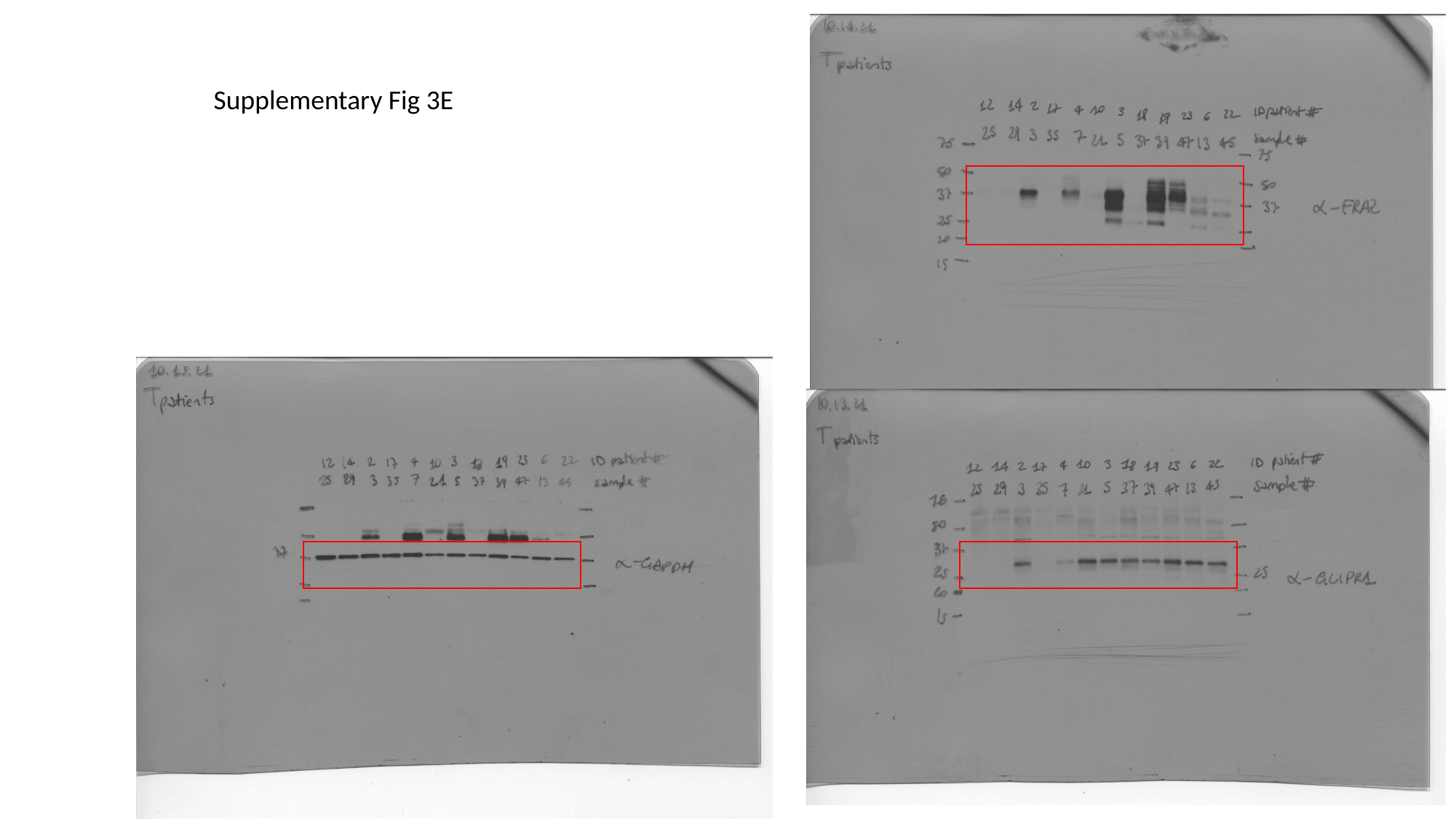

Supplementary Fig 3E

## Slide 5
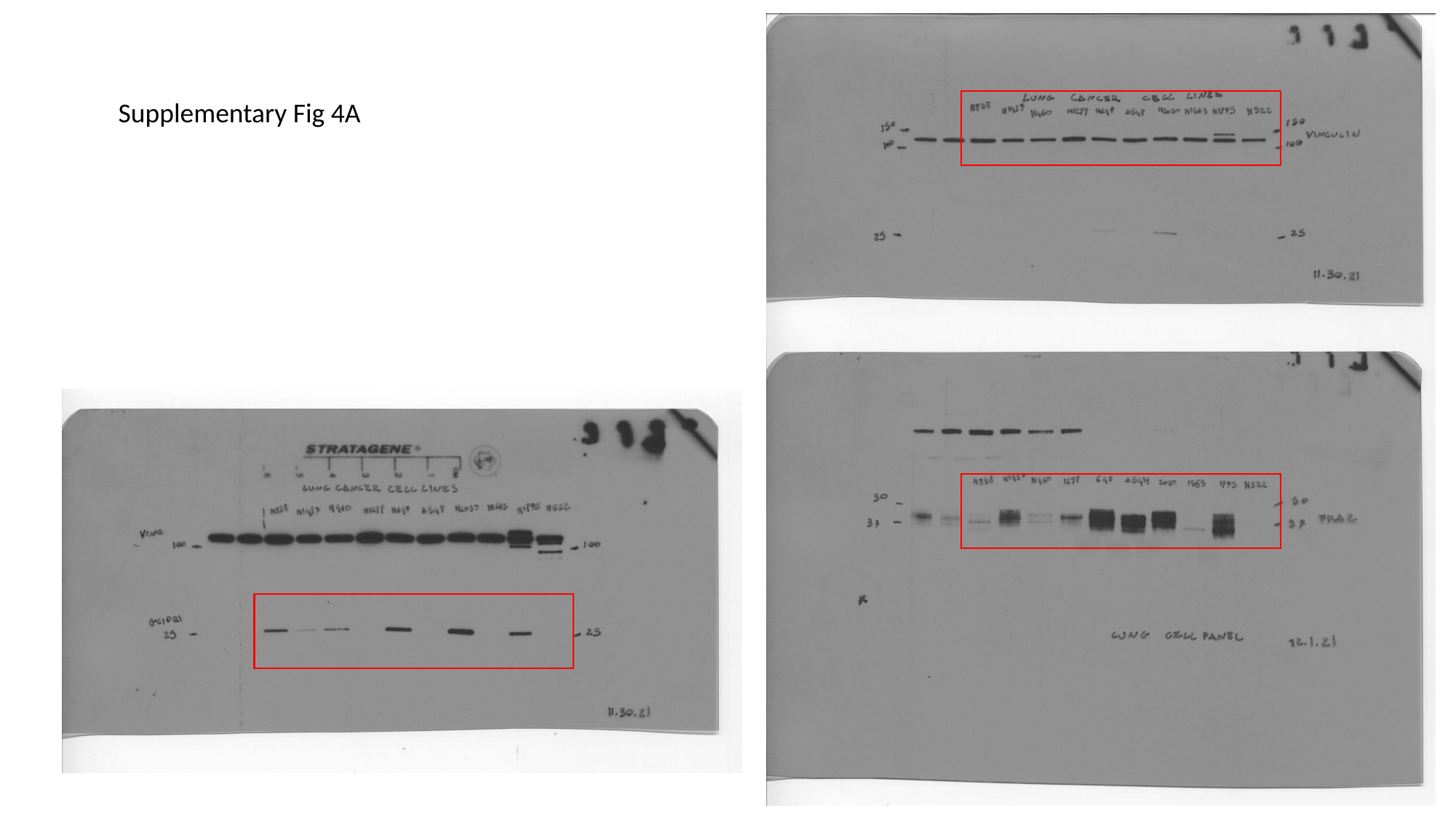

Supplementary Fig 4A

## Slide 6
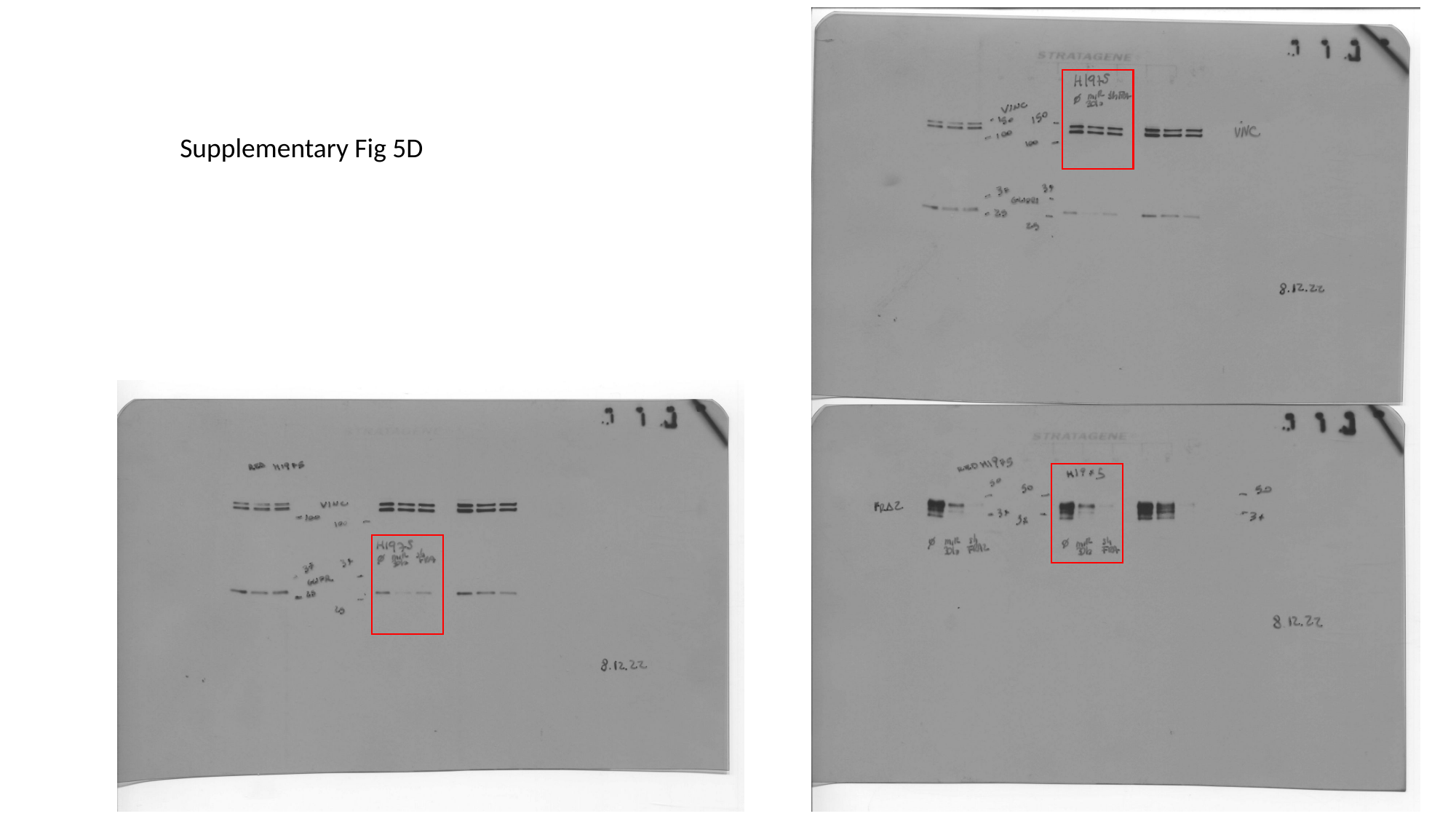

Supplementary Fig 5D

## Slide 7
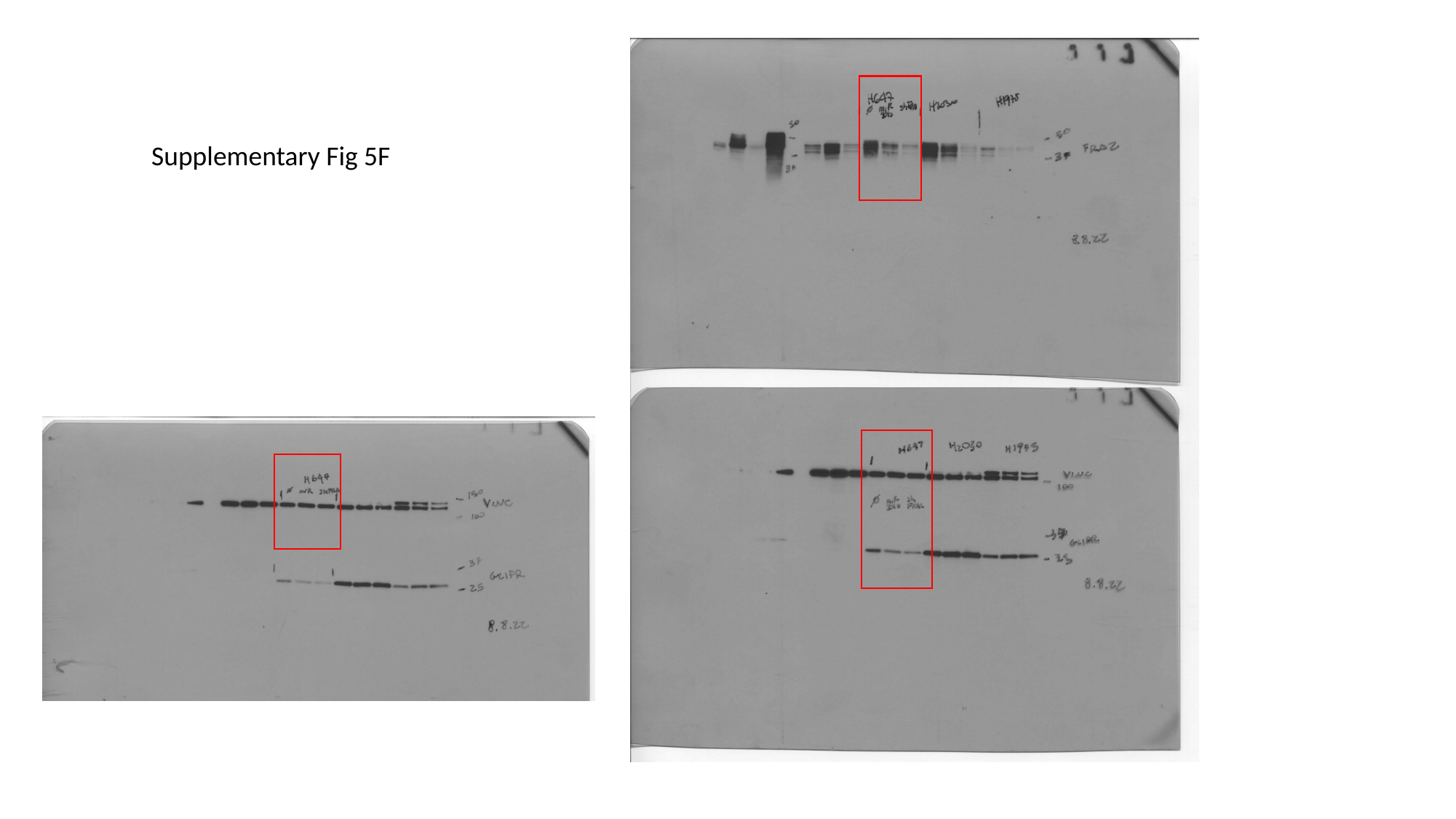

Supplementary Fig 5F

## Slide 8
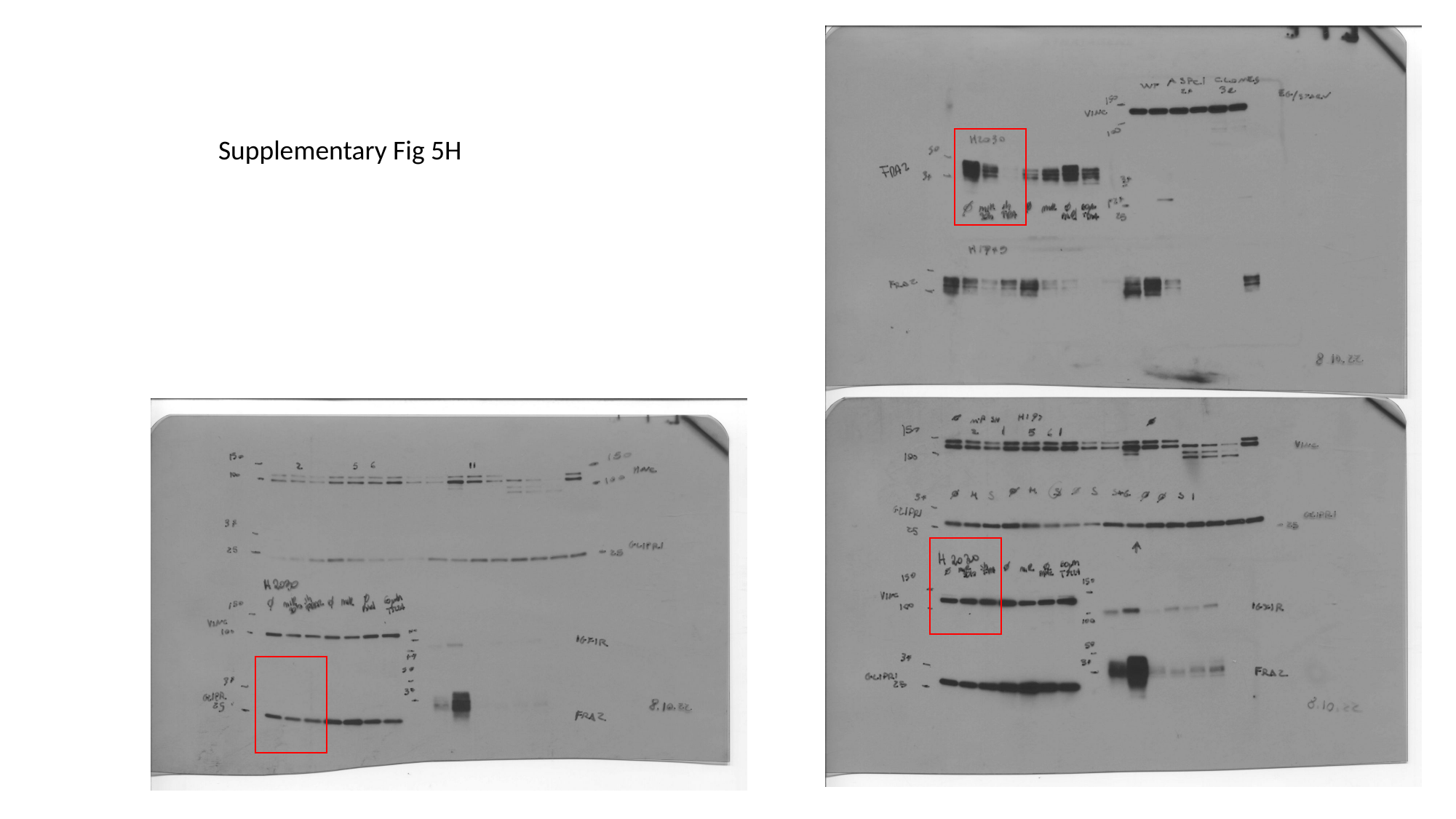

Supplementary Fig 5H

## Slide 9
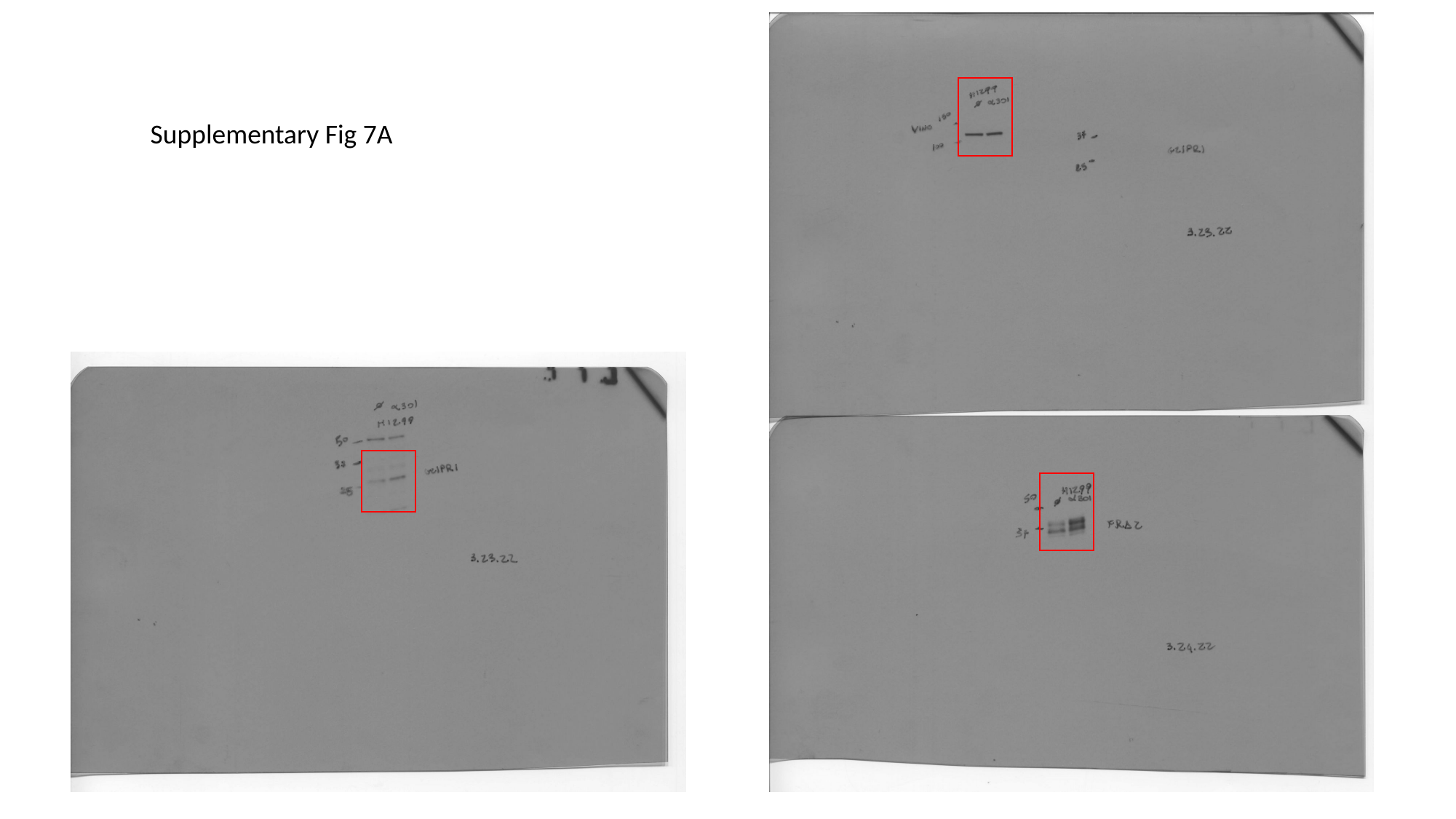

Supplementary Fig 7A

## Slide 10
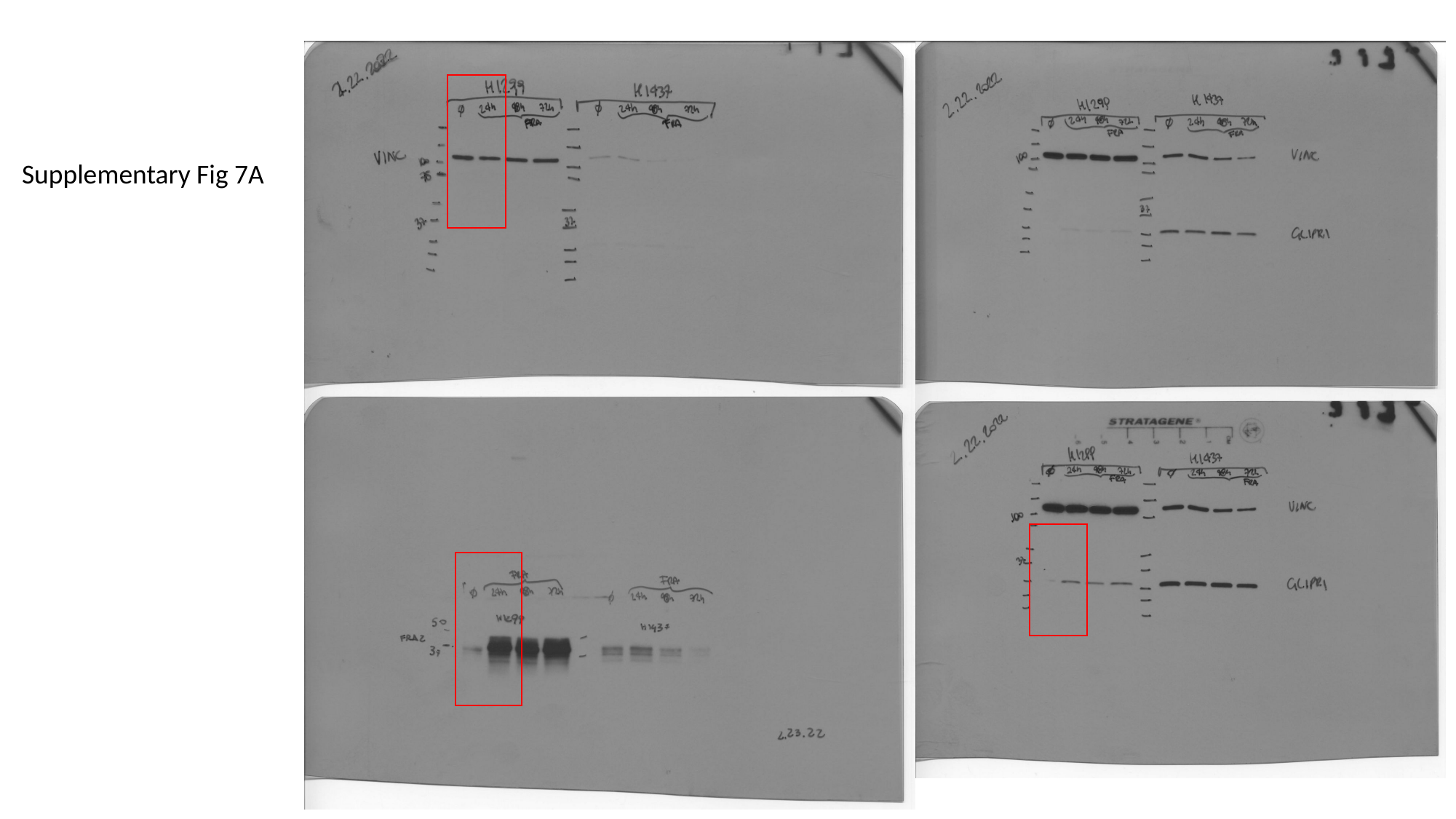

Supplementary Fig 7A

## Slide 11
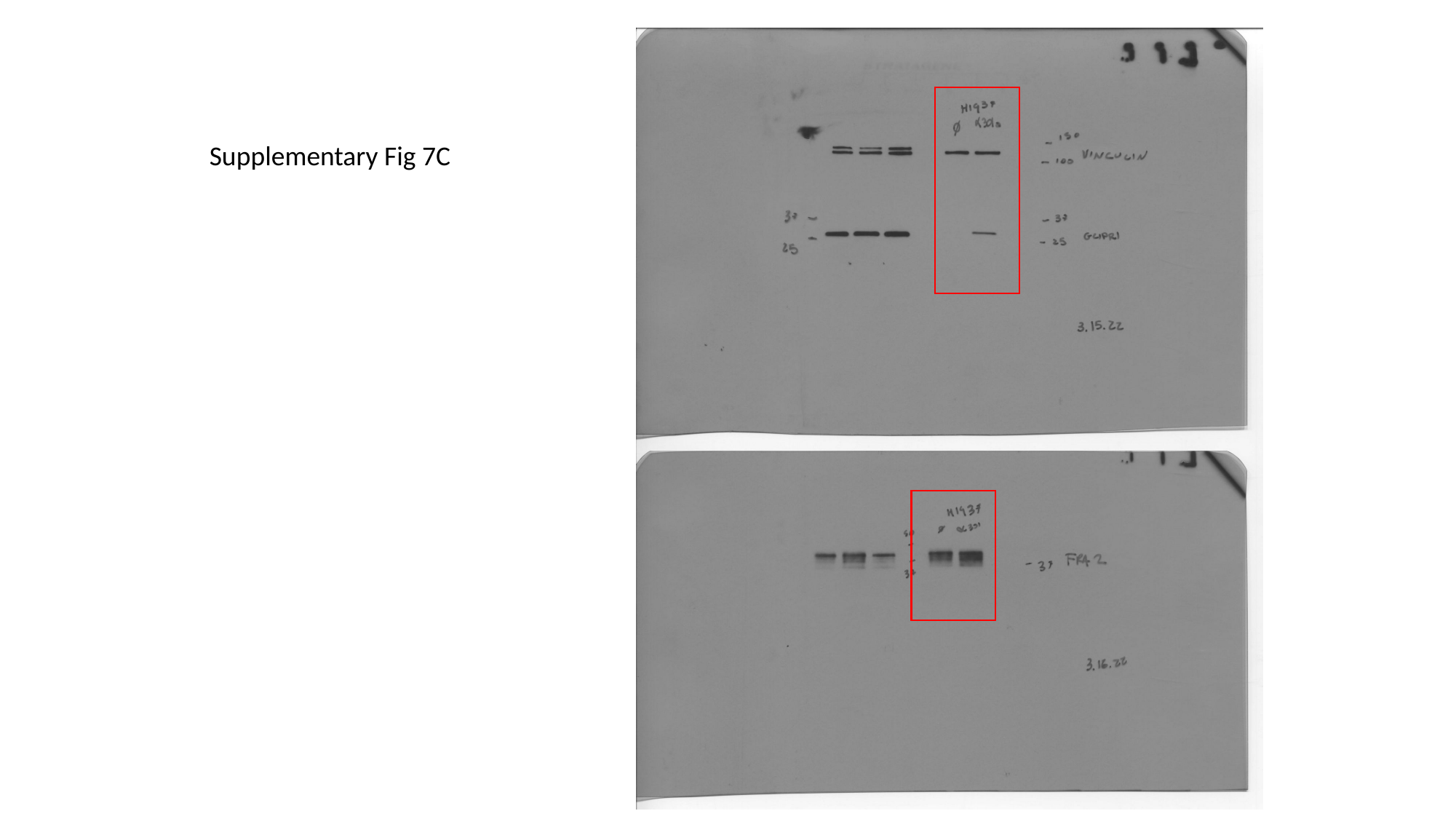

Supplementary Fig 7C

## Slide 12
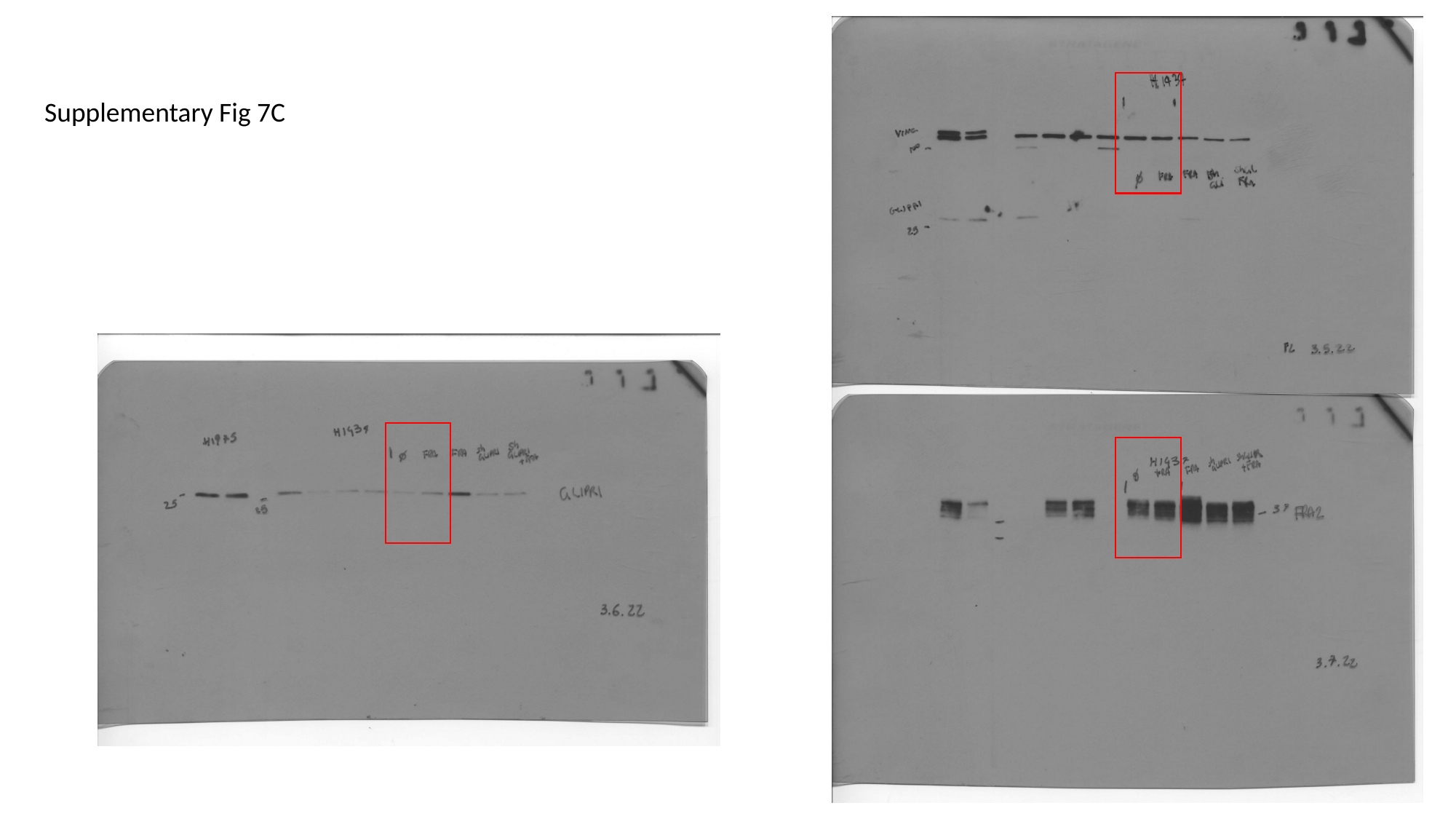

Supplementary Fig 7C

## Slide 13
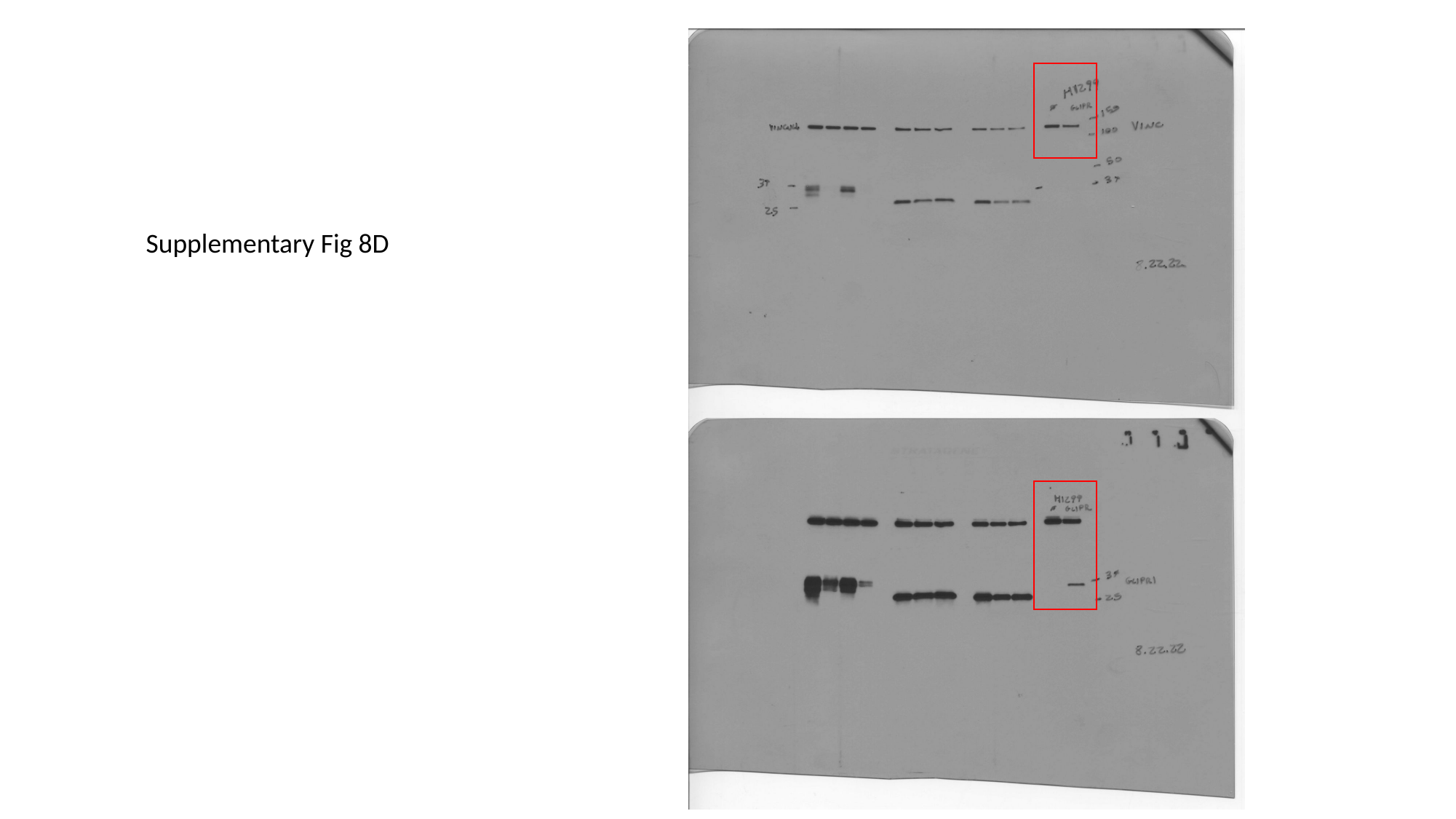

Supplementary Fig 8D

## Slide 14
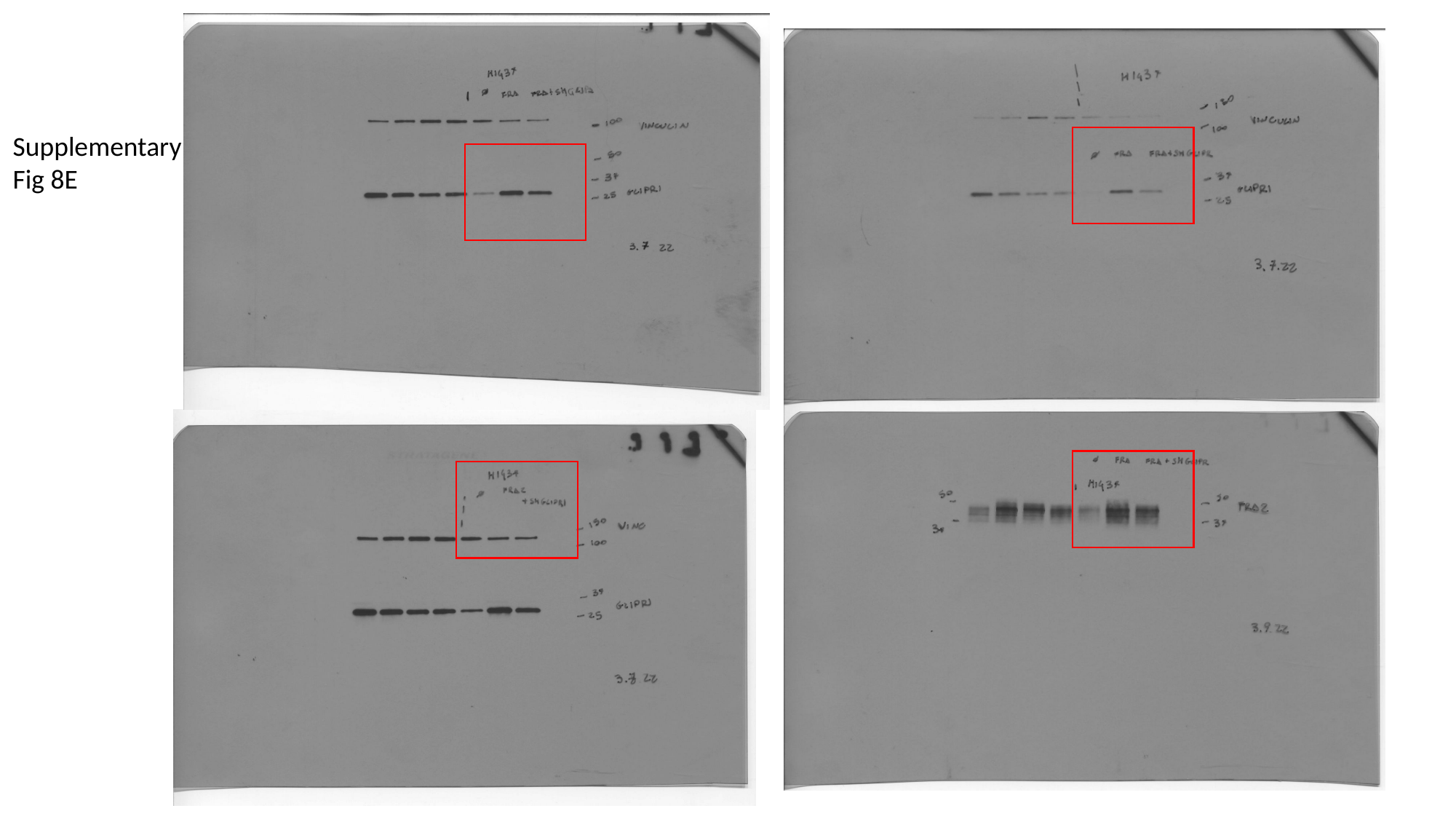

Supplementary
Fig 8E

## Slide 15
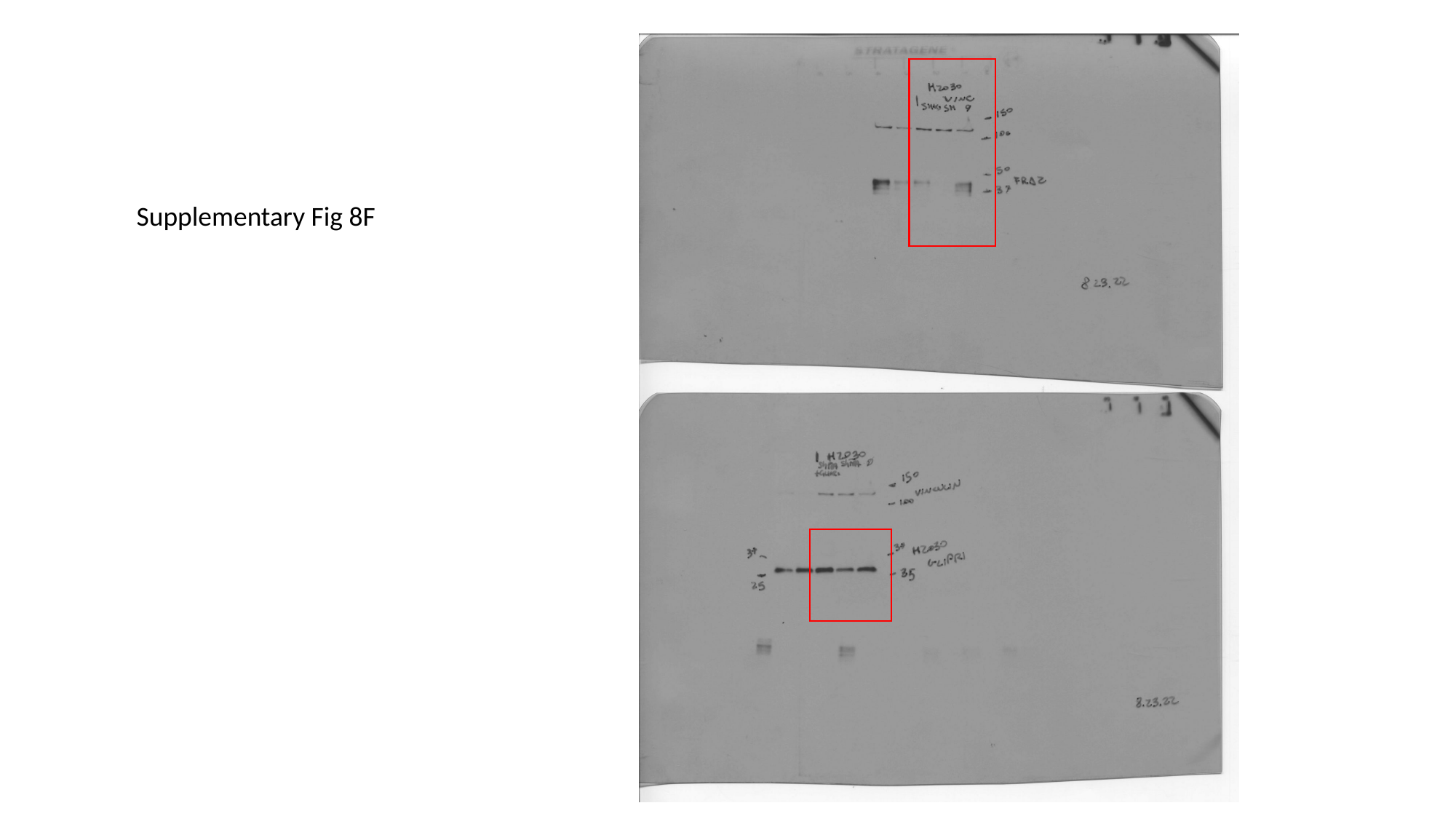

Supplementary Fig 8F
